# Association between OLD and IBD: a systematic review and meta-analysis

**DOI:** 10.3389/fimmu.2026.1726732

**Published:** 2026-06-30

**Authors:** Chaowei Ding, Yunmeng Wang, Tongxinwei Sun, Zexin Liu

**Affiliations:** 1 Department of Respiratory and Critical Care Medicine, Xiamen Humanity Hospital of Fujian Medical University, Xiamen, Fujian, China; 2 Department of Cardiovascular Medicine, The Second Hospital of Hebei Medical University, Shijiazhuang, Hebei, China; 3 Department of Respiratory and Critical Care Medicine, The Second Hospital of Hebei Medical University, Shijiazhuang, Hebei, China

**Keywords:** comorbidity, inflammatory bowel disease, meta-analysis, obstructive lung diseases, systematic review

## Abstract

**Objective:**

This study aimed to systematically evaluate the bidirectional association between obstructive lung diseases (OLD) [including chronic obstructive pulmonary disease (COPD), asthma, and bronchiectasis] and inflammatory bowel disease (IBD) [including Crohn’s disease (CD) and ulcerative colitis (UC)].

**Methods:**

Following the Preferred Reporting Items for Systematic Reviews and Meta-Analyses (PRISMA) guidelines, we conducted a systematic search of the PubMed, Embase, and Cochrane Library databases to identify relevant observational studies published up to 30 June 2025. The included studies were cohort studies, case–control studies, and cross-sectional studies that reported relative risk (RR), hazard ratio (HR), or odds ratio (OR) with 95% confidence intervals (CIs). Study quality was assessed using the Newcastle–Ottawa Scale (NOS), and data were analyzed using random effects or fixed effects models. HRs, RRs, and ORs were analyzed separately according to the direction of association and the disease subtype.

**Results:**

A total of 30 observational studies were included. In the estimate-stratified analyses, IBD was associated with an increased risk of subsequent COPD, while COPD was generally associated with increased risks of subsequent CD and UC, although the COPD-to-CD and COPD-to-UC analyses showed substantial heterogeneity. For bronchiectasis, available evidence suggested a positive association with IBD; however, the pooled estimate for IBD and subsequent bronchiectasis was imprecise and should be interpreted with caution. For asthma, the association was more consistent: IBD was associated with an increased risk of subsequent asthma in both HR- and OR-based analyses, and asthma was also associated with subsequent IBD in the HR-based analysis. The bidirectional association was generally stronger and more consistent for CD than for UC.

**Conclusion:**

This systematic review and meta-analysis supports positive associations between OLD and IBD, particularly for COPD with the IBD subtypes and for asthma with IBD, especially CD. These associations should be interpreted according to the OLD subtype and the age context. Clinicians should be attentive to respiratory comorbidities in patients with IBD, and appropriate respiratory symptom assessment or screening may help early identification and management.

**Systematic Review Registration:**

https://www.crd.york.ac.uk/prospero/, identifier CRD420251169706.

## 1 Introduction

Inflammatory bowel disease (IBD), encompassing Crohn’s disease (CD) and ulcerative colitis (UC), is characterized by chronic, relapsing intestinal inflammation. The global prevalence of IBD currently ranges from 0.3% to 1%, with a steady increase observed (1, 2). The societal burden of IBD is multifaceted, including not only substantial direct medical costs, disease-related disability, and reduced workforce productivity but also the high incidence of extraintestinal complications, which further exacerbate individual suffering and deplete healthcare resources (1). While the clinical use of biologics and small-molecule targeted therapies has significantly altered the course of IBD, a cure remains elusive (3). Early identification of modifiable risk factors is thus critical to delaying disease onset. Previous research has identified genetic predisposition, family history of IBD, smoking, antibiotic use, a monotonous diet, and dysbiosis as independent risk factors for IBD (4–8). In addition, chronic obstructive pulmonary disease (COPD), asthma, and bronchiectasis have been linked to IBD (9–11), suggesting a potential interaction between the respiratory system and intestinal immune homeostasis.

Obstructive lung diseases (OLD) comprise a group of chronic respiratory conditions marked by persistent or recurrent airflow limitation, including COPD, asthma, bronchiectasis, and cystic fibrosis (12). COPD is characterized by an incompletely reversible airflow limitation, which is primarily associated with smoking and exposure to harmful particles (13). Asthma is typically characterized by airway hyperresponsiveness and reversible airflow limitation, often with an allergic background (14). Bronchiectasis involves bronchial wall structural damage, chronic purulent inflammation, and persistent or recurrent expectoration (15). Cystic fibrosis is an autosomal-recessive genetic disorder primarily affecting the exocrine glands, leading to abnormally thick mucus secretion and, when the lungs are affected, chronic airway obstruction and recurrent infections (16). Despite the differing disease features, all OLD subtypes result in progressive lung function decline, heightened risk of acute exacerbations, and increased complication rates. The global patient population has surpassed hundreds of millions. Importantly, emerging evidence suggests a potential link between OLD and IBD in terms of pathogenesis—both share common underlying mechanisms such as immune dysregulation, epithelial barrier dysfunction, and microbiome imbalances, suggesting a bidirectional risk association between the two disease groups (17). Previous studies have primarily examined the relationship between individual airway diseases and IBD: for instance, a 2019 meta-analysis by Labarca et al., which included four retrospective cohort studies, reported a hazard ratio (HR) of 2.02 (95%CI = 1.56–2.63) for new-onset IBD in patients with COPD (18). Kuenzig et al. confirmed through 18 studies that asthma is significantly associated with both CD and UC [relative risk (RR) ≈ 1.30–1.34] (19). However, several critical gaps remain in the current literature: first, the association between bronchiectasis and IBD has not been systematically evaluated. Second, no study has integrated COPD, asthma, and bronchiectasis into a unified analytical framework to explore their bidirectional relationships with IBD. These gaps prevent a holistic understanding of the comorbidity pattern of the two chronic inflammatory diseases from the “gut–lung axis” perspective and limit research on comorbidity mechanisms and clinical screening strategies.

Given these unresolved limitations, a well-designed and comprehensive quantitative study is urgently warranted. Accordingly, this study was specifically designed to address the aforementioned clinical and scientific needs: we systematically searched observational studies published up to 30 June 2025 in the PubMed, Embase, and Cochrane Library databases. Using systematic review and meta-analysis, we simultaneously evaluated the bidirectional associations between COPD, asthma, bronchiectasis, and IBD (including CD and UC) within a unified framework for the first time. We quantitatively pooled association estimates and explored sources of heterogeneity, as well as systematically addressed the identified literature gaps, aiming to clarify the strength and consistency of the associations between OLD and IBD and to provide high-quality evidence for elucidating the gut–lung axis and guiding clinical comorbidity screening and multidisciplinary management.

## 2 Method

### 2.1 Literature retrieval

This study adhered to the guidelines of the Preferred Reporting Items for Systematic Reviews and Meta-Analyses (PRISMA) (20). A protocol has been registered with PROSPERO under registration no. CRD420251169706.

A systematic search was conducted in the PubMed, EMBASE, and Cochrane Library databases from inception to 30 June 2025. The search strategy employed Medical Subject Headings (MeSH) and their variants, including terms such as “obstructive lung disease, COPD, asthma, bronchiectasis, cystic fibrosis” and “inflammatory bowel disease” (detailed search strategy in **Supplementary Table 1**). In addition, the references from the included articles and previous relevant systematic reviews were manually reviewed to identify any missing studies.

### 2.2 Inclusion and exclusion criteria

The inclusion criteria were: 1) cohort studies, case–control studies, or cross-sectional studies; 2) studies investigating the relationship between OLD (COPD, asthma, bronchiectasis, and cystic fibrosis) and IBD; and 3) studies reporting RR, HR, or odds ratio (OR) with 95% confidence intervals (CIs).

The exclusion criteria were: 1) non-human studies, case reports, reviews, letters, meta-analyses, or conference abstracts; 2) multiple publications from the same cohort, with preference given to the study containing the most recent statistical data; 3) studies with inaccessible full text; 4) non-English studies; and 5) cohorts where both the observed and control populations had comorbid conditions that could confound the outcome (e.g., coronary heart disease or heart failure).

### 2.3 Data extraction and quality assessment

Two authors (Ding and Wang) independently screened the abstracts and performed full-text reviews to assess inclusion eligibility. Discrepancies were resolved by consensus with a third author (Liu). The following data were extracted from the included studies: author, year, country, sample size, study design type (prospective cohort, retrospective cohort, cross-sectional, or case–control), baseline patient characteristics, and type of OLD (COPD, asthma, bronchiectasis, or cystic fibrosis). We also extracted the direction of the association, estimate type (HR, RR, or OR), and adjusted covariates and whether the estimate was adjusted or unadjusted. To address the potential influence of age profile and disease onset age on the observed associations, we additionally extracted age-related information from the full text of all included studies. The extracted information included age eligibility criteria; baseline age distribution; age at IBD diagnosis or index date; age at OLD diagnosis or onset when available; pediatric, adolescent, adult, or older adult population definitions; and age-stratified or age-specific association estimates. When studies reported age groups, age-specific incidence rates, or age-related subgroup estimates, these data were summarized systematically Study quality was assessed using the Newcastle–Ottawa Scale (NOS) (21), with studies scoring ≥7 considered high quality.

### 2.4 Data pooling and analysis

The HRs, RRs, and ORs with corresponding 95%CIs were extracted from the included studies. Given the methodological differences among HRs, RRs, and ORs, these estimate types were not converted into a single metric or combined in the primary analyses. Instead, the HRs, RRs, and ORs were analyzed separately according to the direction of association and the disease subtype. When two or more studies reported the same type of estimate for the same exposure–outcome comparison, pooled estimates were calculated using fixed effects or random effects models. When only one study was available for a given comparison or estimate type, the result was presented descriptively without pooling.

Statistical heterogeneity was assessed using the *I*^2^ statistic and Cochran’s *Q* test (22). A random effects model was used when substantial heterogeneity was present (*p* < 0.10 or *I*^2^ > 50%); otherwise, a fixed effects model was applied. Sensitivity analyses were conducted by sequentially omitting individual studies when a sufficient number of studies was available (23). Subgroup analyses were further performed for OR-based asthma-related analyses according to sample size, covariate adjustment, smoking adjustment, socioenvironmental factor adjustment, model complexity, and study design. Socioenvironmental factors included education, income, deprivation index, residence, urbanization, region or municipality, and related socioeconomic or environmental indicators when reported. For subgroup analyses according to model complexity, covariate adjustment was categorized as unadjusted, minimal, moderate, or full adjustment. Unadjusted models were not adjusted for any covariates. Minimal adjustment refers to models adjusted only for age and/or sex. Moderate adjustment refers to models that were adjusted for age and sex plus up to three additional covariates. Full adjustment refers to models that were adjusted for age and sex plus more than three additional covariates, such as comorbidities, lifestyle factors, socioeconomic factors, environmental exposures, or other clinically relevant variables. Because the adjustment strategies varied across studies, these subgroup analyses were considered exploratory and were used mainly to identify potential sources of heterogeneity. As the age categories, the definitions of disease onset, and the age-specific estimates differed substantially across the included studies, formal age-stratified meta-analysis was not performed. Instead, we conducted a systematic narrative synthesis of the age profile and onset age information across included studies. This synthesis was organized according to the OLD subtype, the IBD subtype, the direction of association, and whether the included evidence represented pediatric, adolescent, adult, older-adult, or mixed-age populations. The purpose of this synthesis was to improve the clinical interpretation of the pooled findings and to avoid overgeneralizing associations across OLD subtypes with distinct age-related patterns. Publication bias was assessed using funnel plots and Egger’s test when applicable. All analyses were conducted using STATA statistical software, version 15.1.

## 3 Results

### 3.1 Literature retrieval

**Figure 1:**
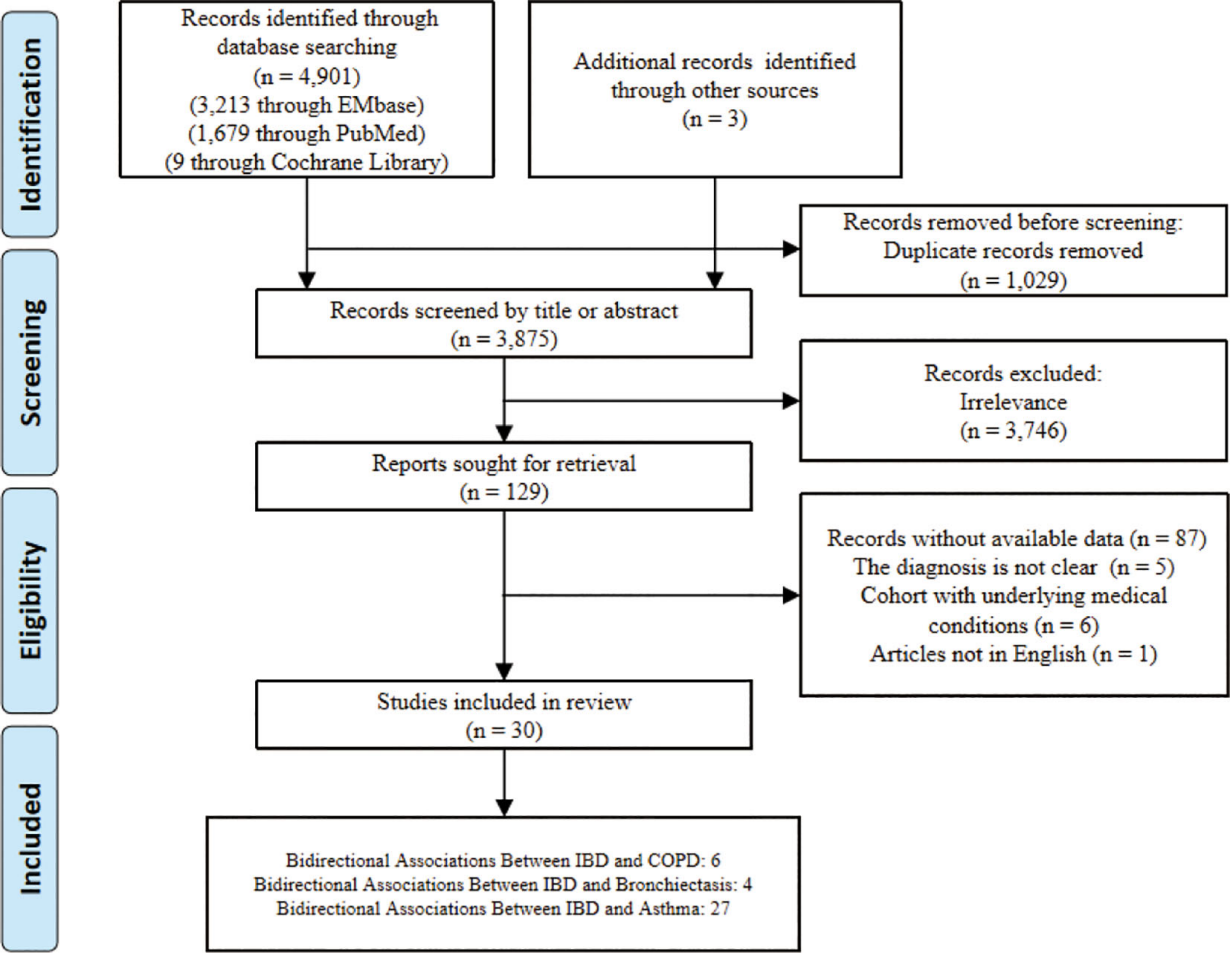
Preferred reporting items for systematic reviews and meta-analyses (PRISMA) flow diagram of the study selection process.

A total of 4,901 articles were identified through database searches, supplemented by three studies obtained from citation reviews, bringing the total to 4,904 articles. After excluding 1,029 duplicates, 3,875 articles remained for initial screening based on the titles and abstracts. Of these, 3,746 were deemed irrelevant and were excluded. The full texts of the remaining 129 articles were reviewed for detailed evaluation, and 30 articles were ultimately included. The study screening process is illustrated in **Figure 1**. Among these 30 included studies, six reported associations between IBD and COPD (10, 24–28), including two studies describing the association between IBD and subsequent COPD, two describing the association between COPD and subsequent IBD, four describing the association between COPD and subsequent CD, and four describing the association between COPD and subsequent UC. There were four studies that reported the association between bronchiectasis and IBD (9, 10, 28, 29), including three studies describing the association between IBD and subsequent bronchiectasis and one describing the association between bronchiectasis and subsequent IBD. There were 27 studies that explored the mutual relationship between IBD and asthma (9, 10, 24, 26, 28, 30–51), including 12 studies describing the association between IBD and subsequent asthma, five describing the association between asthma and subsequent IBD, 10 describing the association between CD and subsequent asthma, eight describing the association between asthma and subsequent CD, 11 describing the association between UC and subsequent asthma, and seven describing the association between asthma and subsequent UC. Unfortunately, no studies investigating the association between cystic fibrosis and the risk of IBD were identified. Detailed study characteristics are provided in **Tables 1**–**3**.

**Table 1:** Characteristics of the included studies investigating the association between chronic obstructive pulmonary disease (COPD) and inflammatory bowel disease (IBD).

Author, year	Country	Population, n	Gender, female (%)	Age (years)	Study design	Follow-up	COPD diagnosis	IBD diagnosis	IBD type	Direction of association	Multivariable adjustment	NOS
Dan, 2025 (24)	UK	UK Biobank, 430,414	54.1	57.06 ± 8.08	Prospective study	The median follow-up was 11.9 years.	Based on ICD codes	Based on ICD codes, self-reported	IBD, CD, UC	IBD → COPD CD → COPD UC → COPD	Age, age square, sex, BMI, ethnicity, education, Townsend deprivation index, smoking status, degree of physical activity, PM10, numbers of household smokers	8
Jacobsen, 2024 (10)	Denmark	245,515	46.5	42.6 ± 19.5	Retrospective study	The median follow-up was 9.7 years.	Based on ICD codes and medication record	Based on ICD codes	IBD	IBD → COPD	Sex, age group, and year of cohort entry	9
Lee, 2019 (25)	South Korea	7,818,126	56	57.1 ± 10.7	Retrospective study	The average follow-up period was 3.9 ± 1.4 years.	Based on ICD codes and medication record	Based on ICD and RID-V codes	IBD, CD, UC	COPD → IBD COPD → CD COPD → UC	Age, sex, place of resident, income, diabetes mellitus, hypertension, dyslipidemia	9
Brassard, 2015 (26)	Canada	143,904	53.19	NA	Retrospective study	The average follow-up period was 5.2 ± 3.6 years.	≥3 prescriptions for respiratory medication within 1 year, on at least two separate occasions; the third prescription must have occurred at ≥41 years of age.	Based on ICD codes	CD, UC	COPD → CD COPD → UC	Age, sex	8
Ekbom, 2008 (27)	Sweden	180,239	NA	NA	Matched case–control study	15 years	Based on ICD codes	Based on ICD codes	CD, UC	COPD → CD COPD → UC	Age, sex. and county were identified.	9
Raj,2008 (28)	UK	588	NA	NA	Retrospective cohort study	NA	A consistent clinical picture with evidence of airflow obstruction (FEV1 <70% of the predicted and FEV1/FVC <70%). No significant improvement in FEV1 after 200 μg inhaled salbutamol (<15%, or if FEV1<1l, <200 ml improvement).	Based on medical records	IBD, CD, UC	COPD → IBD COPD → CD COPD → UC	NA	6

UC, ulcerative colitis; CD, Crohn’s disease; BMI, body mass index; ICD, International Classification of Diseases; RID-V, Respiratory Infection Disease—Viral; FEV1, forced expiratory volume in 1 s; NA, not available; NOS, Newcastle–Ottawa Scale.

**Table 2:** Characteristics of the included studies investigating the association between bronchiectasis and inflammatory bowel disease (IBD).

Author, year	Country	Population, n	Gender, female (%)	Age (years)	Study design	Follow-up	Bronchiectasis diagnosis	IBD diagnosis	IBD type	Direction of association	Multivariable adjustment	NOS
Lee, 2024 (29)	South Korea	32,565	50.3	47.14 ± 15.3	Retrospective study	The average follow-up period was 9.6 years.	Based on ICD codes	Based on ICD and RID-V codes	IBD, CD, UC	IBD → bronchiectasis CD → bronchiectasis UC → bronchiectasis	Insurance type, body mass index, smoking status, alcohol consumption, physical activity, CCI (Charlson comorbidity index), and medications	9
Jacobsen, 2024 (10)	Denmark	245,515	46.5	42.6 ± 19.5	Retrospective study	The median follow-up was 9.7 years.	Based on ICD codes and medication record	Based on ICD codes	IBD	IBD → bronchiectasis	Sex, age group, and year of cohort entry	9
Pemmasani, 2022 (9)	USA	87,506	57.1	51.9 ± 19.5	Retrospective study	NA	Based on ICD codes	Based on ICD codes	IBD	IBD → bronchiectasis	Age, sex, smoking history	8
Raj, 2008 (28)	UK	215	NA	NA	Retrospective cohort study	NA	Consistent clinical picture with CT evidence of abnormal bronchial dilatation	Based on medical records	IBD, CD, UC	Bronchiectasis → IBD bronchiectasis → CD bronchiectasis → UC	NA	6

UC, ulcerative colitis; CD, Crohn’s disease; ICD, International Classification of Diseases; RID-V, Respiratory Infection Disease—Viral; NA, not available; NOS, Newcastle–Ottawa Scale.

**Table 3:** Characteristics of the included studies on asthma and inflammatory bowel disease (IBD).

Author, year	Country	Population, n	Gender, female (%)	Age (years)	Study design	Follow-up	Asthma diagnosis	IBD diagnosis	IBD type	Direction of association	Multivariable adjustment	NOS
Dan, 2025 (24)	UK	UK Biobank, 430,414	54.1	57.06 ± 8.08	Prospective study	The median follow-up was 11.9 years.	Based on ICD codes	Based on ICD codes, self-reported	IBD, UC, CD	IBD → asthma CD → asthma UC → asthma	Age, age square, sex, BMI, ethnicity, education, Townsend deprivation index, smoking status, degree of physical activity, PM10, number of household smokers	8
Gong, 2024 (30)	Sweden	2,873,445	48.6	NA	Prospective study	35 years	Based on ICD codes and medication record	Based on ICD codes	IBD	IBD → asthma	Sex, parity, and birth year	9
Jacobsen, 2024 (10)	Denmark	245,515	46.5	42.6 ± 19.5	Retrospective study	The median follow-up was 9.7 years.	Based on ICD codes and medication record	Based on ICD codes	IBD	IBD → asthma	Sex, age group, and year of cohort entry	9
Kisiel, 2023 (31)	Sweden	12,155	53.1	51.6 ± 7.2	Cross-sectional study	2 years	Based on medication record and self-reported	Self-reported	IBD, UC, CD	IBD → asthma CD → asthma UC → asthma	Age, sex, BMI, smoking history, educational level, exercise, and center	8
Liljendahl, 2022 (32)	Denmark	366,200	48.6	NA	Prospective study	15–20 years	Based on medication record	Based on ICD codes	IBD, UC, CD	IBD → asthma Asthma → IBD Asthma → CD Asthma → UC	Sex and adjusted for birth year, socioeconomic status, maternal age, birth weight, cesarean section, familiar occurrence of T1D/IBD or asthma	9
Pemmasani, 2022 (9)	USA	87,506	57.1	51.9 ± 19.5	Retrospective study	NA	Based on ICD codes	Based on ICD codes	IBD	IBD → asthma	Age, sex, smoking history	8
Lo, 2021 (33)	USA	101,019	100	NA	Prospective study	22 years	Self-reported	Self-reported and reviewed by gastroenterologists.	UC, CD	Asthma → CD Asthma → UC	Age, study period, race, area of residence, median family income, family history of inflammatory bowel disease, family history of immune-mediated disease, smoking, BMI, physical activity, total fiber intake, NSAID use, oral contraceptive pill, menopausal hormone therapy	9
Soh, 2021 (34)	South Korea	NA	NA	NA	Prospective cohort study	The average follow-up period was 7.3 years.	Based on ICD codes	Based on ICD codes	UC, CD	Asthma → CD Asthma → UC	Age, sex, residence, smoking history, alcohol consumption, regular exercise, income, body mass index, diabetes, hypertension, and dyslipidemia	9
Ghersin, 2020 (35)	Israel	114,213	NA	NA	Retrospective study	NA	Based on medical records	Pathological and imaging diagnosis	UC, CD	UC → asthma	Age, sex, and comorbidities	7
Krishna, 2019 (36)	UK	2,782,348	51.6	37.08 ± 21.08	Retrospective study	The median follow-up was 5.51 years.	Based on ICD codes	Based on ICD codes	IBD	Asthma → IBD	Age, sex, BMI, ethnicity, Townsend deprivation quintile, and smoking status	7
Wasielewska, 2019 (51)	Poland	120	49.2	14.78 ± 3.17	Retrospective study	NA	Based on allergological diagnosis	Pathological and imaging diagnosis	UC, CD	CD → asthma UC → asthma	NA	5
Burisch, 2019 (37)	Denmark	11,777	NA	NA	Prospective cohort study	The average follow-up period was 5.4 years.	Based on ICD codes	Based on ICD codes	UC, CD	IBD → asthma CD → asthma UC → asthma	Age and sex	9
Halling, 2017 (38)	Denmark	140,164	54.7	NA	Cross-sectional study	NA	Based on ICD codes	Based on ICD codes	IBD, UC, CD	IBD → asthma CD → asthma UC → asthma	Sex, age, and municipality	7
Kuenzig, 2017 (39)	Canada	408,264	51.1	NA	Retrospective study	NA	Based on ICD codes	Based on ICD codes	UC, CD	Asthma → CD Asthma → UC	Age, sex, income quintile, age at diagnosis. and living in a metropolitan city	8
Peng, 2015 (40)	China	26,300	52.9	46.41 ± 16.03	Retrospective study	The median follow-up >8 years.	Based on ICD codes and medication record	Based on ICD codes	IBD, UC, CD	IBD → asthma CD → asthma UC → asthma	Age, sex, and other comorbidities (rhinitis, chronic sinusitis, atopic dermatitis, and chronic obstructive pulmonary disease)	8
Brassard, 2015 (26)	Canada	136,178	66.34	NA	Retrospective study	The average follow-up period was 6.5 ± 4 years.	≥3 prescriptions for respiratory medication within 1 year, on at least two separate occasions. The third prescription must have occurred at ≤40 years of age.	Based on ICD codes	UC, CD	Asthma → CD Asthma → UC	Age, sex	8
Virta, 2013 (41)	Finland	2,975	43.11	NA	Matched case–control study	NA	Based on ICD codes	Based on endoscopy and histologic verification	UC, CD	Asthma → CD Asthma → UC	Date of birth, sex, and place of residence	8
Gearry, 2012	New Zealand	1,891	52.8	NA	Matched case–control study	NA	Based on predetermined criteria	Based on predetermined criteria	UC, CD	Asthma → CD Asthma → UC	Family history, cigarette smoking, age category, sex, ethnicity, and social class at birth	7
Yun, 2012 (43)	USA	7,176	43.3	15.1 ± 20.5	Retrospective study	The median follow-up was 5.8 years.	Based on predetermined criteria for asthma (not ICD codes or self-report)	Based on ICD and Berkson codes	IBD	Asthma → IBD	Age, sex	8
Boneberger, 2012 (44)	Germany	226	64.2	26.48 ± 8.22	Case–control study	NA	Based on ICD codes	Based on ICD codes	UC	UC → asthma	Age and sex	7
Kappelman, 2011 (45)	USA	4,595	45.5	15.0 ± 3.47	Matched case–control study	NA	Based on ICD codes	Based on ICD codes or claim	Cannot be classified	CD → asthma UC → asthma	Age, sex, health plan type, and geographic region	8
Haapamäki, 2011 (46)	Finland	8,493	64.0	44.1 ± 13.4	Matched case–control study	NA	Based on medical records	Based on medical records	IBD	IBD → asthma	Age, gender, and hospital area	7
Sibtain, 2011 (47)	Canada	216	NA	NA	Prospective cohort study	NA	Self-reported	Based on clinical, radiological, and endoscopic evidence	IBD, UC, CD	IBD → asthma CD → asthma UC → asthma	Age and sex	7
Fenta, 2010 (48)	USA	462	49.6	33.6 ± 16.3	Matched case–control study	NA	Based on predetermined criteria	Based on predetermined criteria	IBD	Asthma → IBD	Age, sex	8
Raj, 2008 (28)	UK	893	NA	NA	Retrospective cohort study	NA	A consistent clinical picture with objective evidence of variable outflow obstruction and/or airway hyperresponsiveness (requiring at least one of the following: >15% rise in FEV1–20 min after 200 μg inhaled salbutamol, >20% maximum PEF variability obtained from twice daily PEF over 14 days, a positive methacholine provocation test [PC20 <8 mg/ml])	Based on medical records	IBD, UC, CD	Asthma → IBD Asthma → CD Asthma → UC	NA	6
Weng, 2007 (49)	USA	663,005	47.5	NA	Cross-sectional study/matched case–control study	NA	Based on ICD codes	Based on ICD codes	IBD, UC, CD	IBD → asthma CD → asthma UC → asthma	Age, gender, length of enrollment, smoking	8
Myrelid, 2004 (50)	Sweden	1,059	52.7	NA	Matched case–control study	NA	Self-reported	Self-reported	CD	CD → asthma	Age, gender, place of residence, and coexistence of any other atopic manifestation	8

UC, ulcerative colitis; CD, Crohn’s disease; BMI, body mass index; ICD, International Classification of Diseases; FEV1, forced expiratory volume in 1 s; NA, not available; NOS, Newcastle–Ottawa Scale.

### 3.2 Quality assessment

The quality of the included studies was assessed using the NOS. The majority of the studies were of high quality (NOS score ≥7.0), while two studies were considered of moderate quality (NOS score 5–6) (28, 51). The quality scores of the included studies are presented in **Tables 1**-**3**.

### 3.3 Data analysis

#### 3.3.1 Associations between IBD and COPD

**Figure 2:**
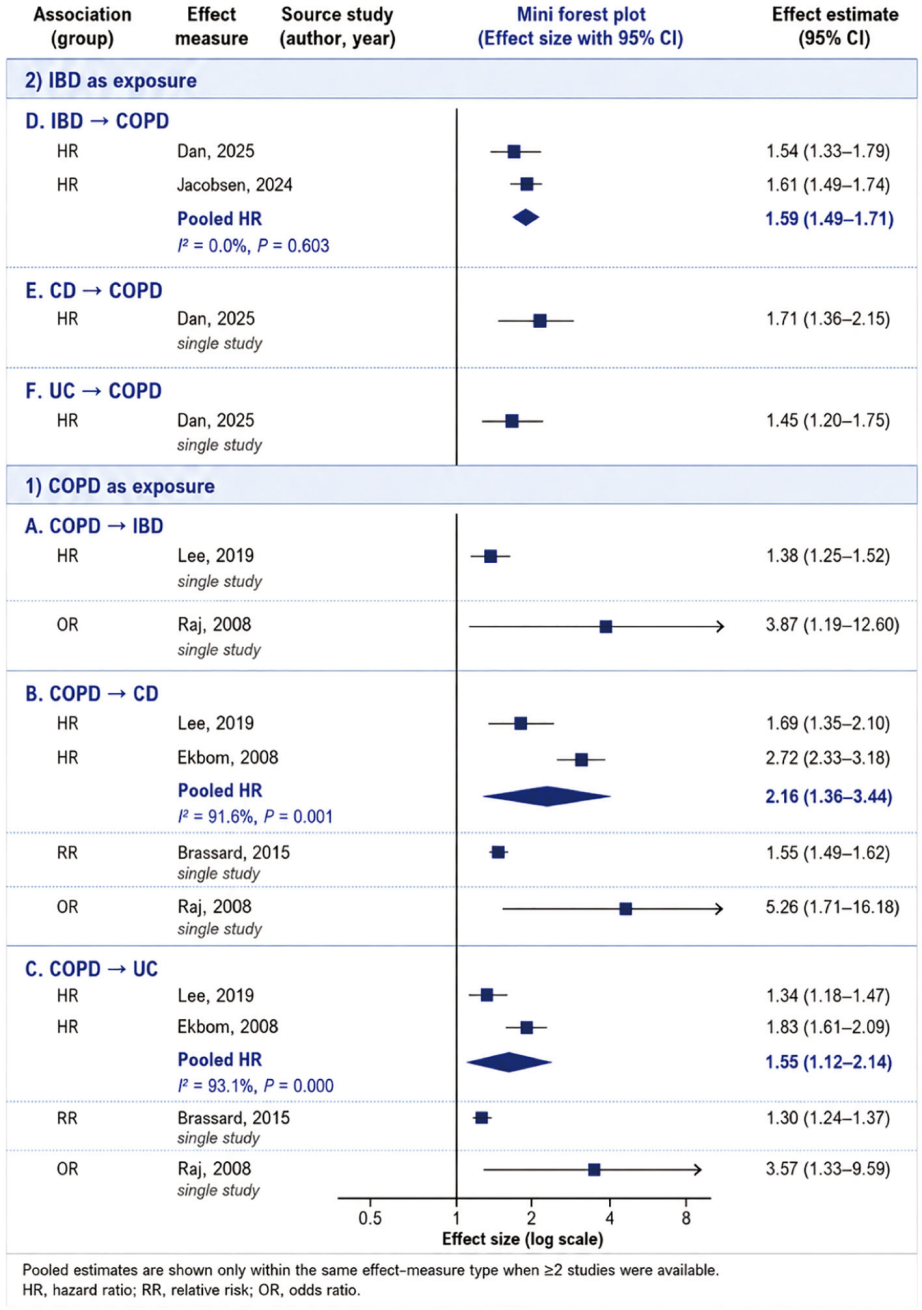
Summary of the bidirectional associations between chronic obstructive pulmonary disease (COPD) and inflammatory bowel disease (IBD), stratified by estimate type.

The bidirectional associations between COPD and IBD are summarized in **Figure 2**.

For the association from IBD to subsequent COPD, two studies reported HRs. Dan et al. (24) reported an HR of 1.54 (95%CI = 1.33–1.79), and Jacobsen et al. (10) reported an HR of 1.61 (95%CI = 1.49–1.74). The pooled HR was 1.59 (95%CI = 1.49–1.71), with no observed heterogeneity (*I*^2^ = 0.0%, *p* = 0.603). For the IBD subtypes, Dan et al. reported an HR of 1.71 (95%CI = 1.36–2.15) for CD and subsequent COPD and an HR of 1.45 (95%CI = 1.20–1.75) for UC and subsequent COPD. Because each subtype-specific comparison was based on a single study, no pooled estimate was generated.

For the reverse direction, one HR-based study and one OR-based study evaluated COPD and subsequent IBD. Lee et al. (25) reported an HR of 1.38 (95%CI = 1.25–1.52), whereas Raj et al. (28) reported an OR of 3.87 (95%CI = 1.19–12.60). These estimates were not pooled because they were based on different estimate types.

For COPD and subsequent CD, two studies reported HRs. Lee et al. (25) reported an HR of 1.69 (95%CI = 1.35–2.10), and Ekbom et al. (27) reported an HR of 2.72 (95%CI = 2.33–3.18). The pooled HR was 2.16 (95%CI = 1.36–3.44), with substantial heterogeneity (*I*^2^ = 91.6%, *p* = 0.001). One RR-based study by Brassard et al. (26) reported an RR of 1.55 (95%CI = 1.49–1.62), and one OR-based study by Raj et al. (28) reported an OR of 5.26 (95%CI = 1.71–16.18). These single-study RR and OR estimates are presented descriptively and were not combined with the HR-based pooled estimate.

For COPD and subsequent UC, two studies reported HRs. Lee et al. (25) reported an HR of 1.34 (95%CI = 1.18–1.47), and Ekbom et al. (27) reported an HR of 1.83 (95%CI = 1.61–2.09). The pooled HR was 1.55 (95%CI = 1.12–2.14), with substantial heterogeneity (*I*^2^ = 93.1%, *p* < 0.001). Brassard et al. (26) reported an RR of 1.30 (95%CI = 1.24–1.37), and Raj et al. (28) reported an OR of 3.57 (95%CI = 1.33–9.59). These single-study RR and OR estimates are presented descriptively and were not combined with the HR-based pooled estimate. Overall, the available estimates generally suggest positive associations between COPD and IBD-related outcomes, although the COPD-to-CD and COPD-to-UC pooled HR analyses should be interpreted with caution because they were based on only two studies and showed high heterogeneity.

#### 3.3.2 Associations between IBD and bronchiectasis

**Figure 3:**
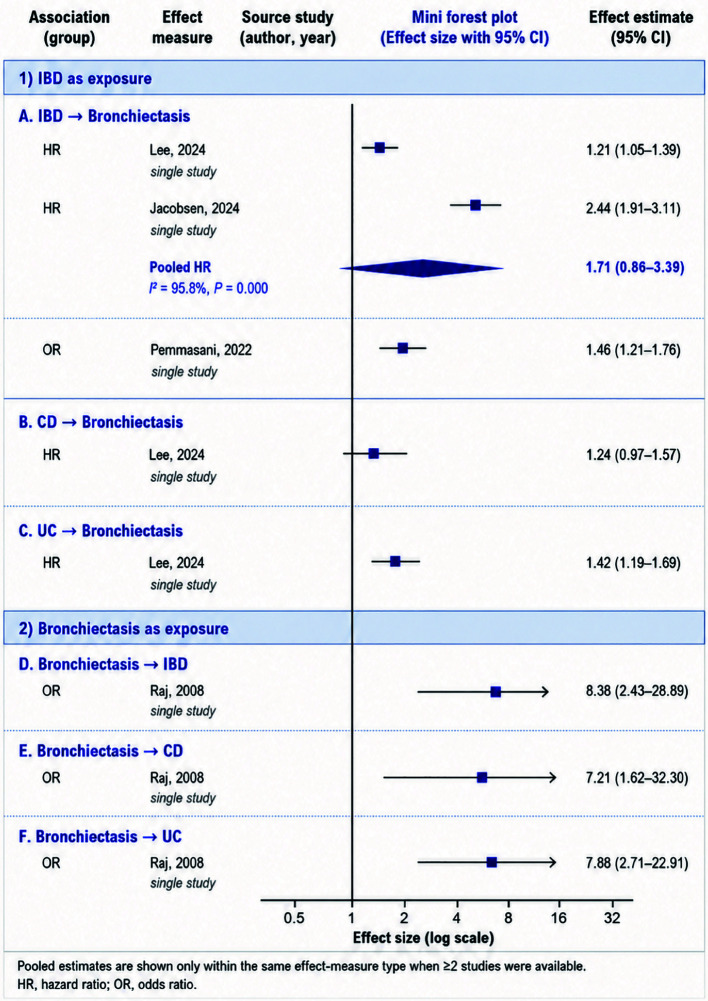
Summary of the bidirectional associations between inflammatory bowel disease (IBD) and bronchiectasis, stratified by estimate type.

The associations between bronchiectasis and IBD are summarized in **Figure 3**.

For IBD and subsequent bronchiectasis, two studies reported HRs. Lee et al. (29) reported an HR of 1.21 (95%CI = 1.05–1.39), and Jacobsen et al. (10) reported an HR of 2.44 (95%CI = 1.91–3.11). The pooled HR was 1.71 (95%CI = 0.86–3.39), with substantial heterogeneity (*I*^2^ = 95.8%, *p* < 0.001). Although both individual HR estimates were above the null value, the pooled HR was imprecise and crossed the null value. One OR-based study by Pemmasani et al. (9) reported an OR of 1.46 (95%CI = 1.21–1.76), which is presented as a single-study estimate and was not combined with the HR-based pooled estimate.

For the IBD subtype analyses, Lee et al. (29) reported an HR of 1.24 (95%CI = 0.97–1.57) for CD and subsequent bronchiectasis and an HR of 1.42 (95%CI = 1.19–1.69) for UC and subsequent bronchiectasis. Because each subtype-specific comparison was based on a single study, no pooled estimate was generated.

For the reverse direction, only one study was available. Raj et al. (28) reported ORs of 8.38 (95%CI = 2.43–28.89) for bronchiectasis and IBD, 7.21 (95%CI = 1.62–32.30) for bronchiectasis and CD, and 7.88 (95%CI = 2.71–22.91) for bronchiectasis and UC. These estimates suggested positive associations, but should be interpreted as exploratory because they were derived from a single study.

#### 3.3.3 Associations between IBD and asthma

##### 3.3.3.1 Associations between overall IBD and asthma

**Figure 4:**
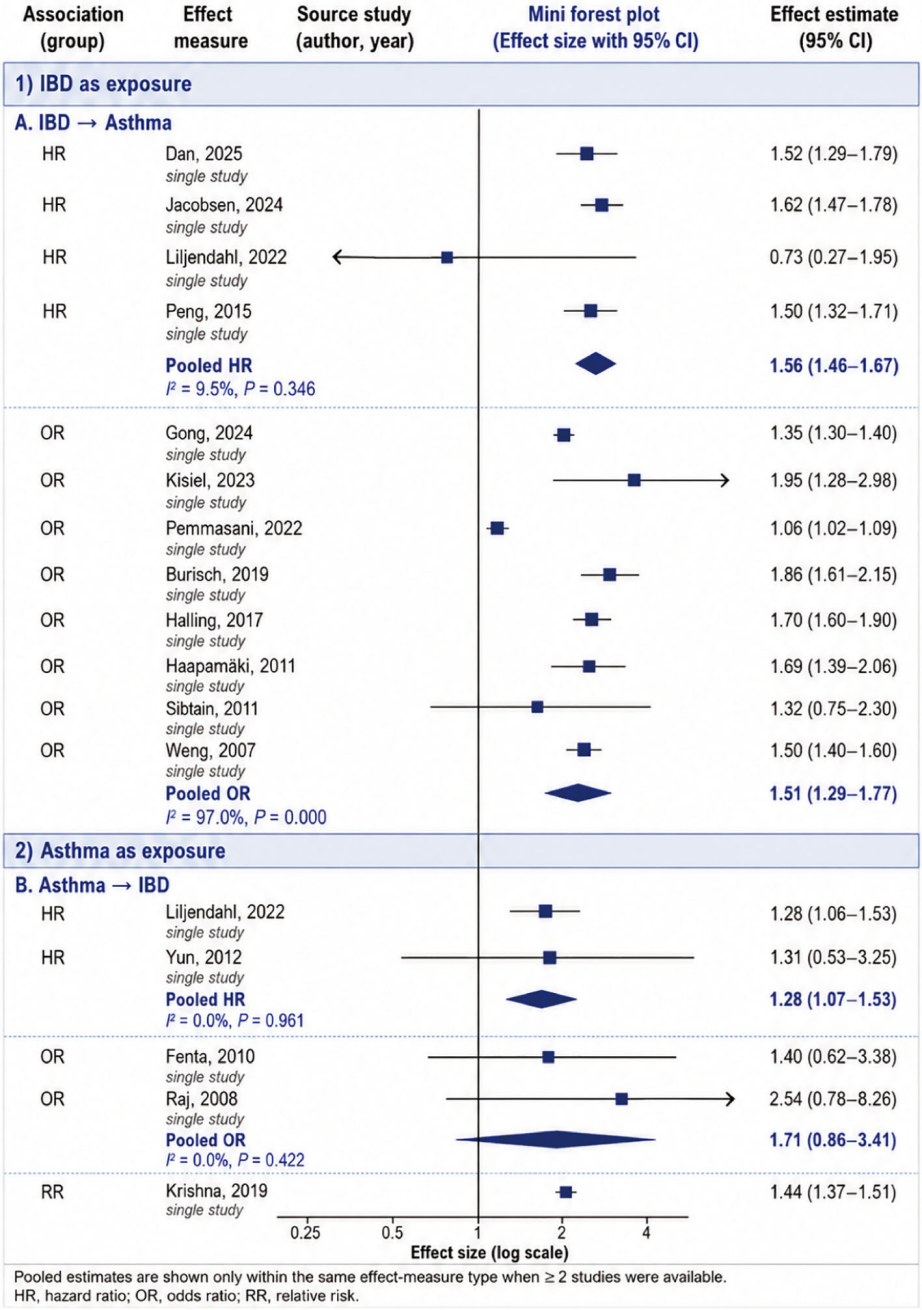
Summary of the bidirectional associations between inflammatory bowel disease (IBD) and asthma, stratified by estimate type.

The bidirectional associations between IBD and asthma are summarized in **Figure 4**. Given that different studies reported different types of estimates, the HRs, ORs, and RRs were summarized and analyzed separately.

For the association between IBD and subsequent asthma, four studies reported HR estimates. The HRs reported by Dan et al. (24), Jacobsen et al. (10), Liljendahl et al. (32), and Peng et al. (40) were 1.52 (95%CI = 1.29–1.79), 1.62 (95%CI = 1.47–1.78), 0.73 (95%CI = 0.27–1.95), and 1.50 (95%CI = 1.32–1.71), respectively. The pooled HR was 1.56 (95%CI = 1.46–1.67), with low heterogeneity (*I*^2^ = 9.5%, *p* = 0.346), suggesting that IBD was associated with an increased risk of subsequent asthma.

There were eight studies that reported OR estimates for the association between IBD and subsequent asthma. The pooled OR was 1.51 (95%CI = 1.29–1.77), indicating a positive association. However, substantial heterogeneity was observed (*I*^2^ = 97.0%, *p* < 0.001), suggesting considerable differences across studies. The sensitivity analysis showed that the study by Pemmasani et al. (9) had a notable influence on the pooled estimate. After excluding this study, the pooled OR remained significant at 1.60 (95%CI = 1.43–1.79), although heterogeneity remained high (*I*^2^ = 86.4%, *p* < 0.001) (**Supplementary Figure 1A**). Therefore, further subgroup analyses were performed according to sample size, smoking adjustment, socioenvironmental factor adjustment, model complexity, and study design (**Supplementary Table 2**). These analyses provided important insights into the source of heterogeneity. Heterogeneity was substantially lower among studies that adjusted for smoking than among those without smoking adjustment (*I*^2^ = 31.9% *vs*. 90.1%). A similar pattern was observed for socioenvironmental adjustment: studies adjusting for socioenvironmental factors showed no within-subgroup heterogeneity (*I*^2^ = 0.0%), whereas studies without such adjustment retained high heterogeneity (*I*^2^ = 86.8%). Model complexity showed a consistent pattern, with moderately adjusted models showing lower heterogeneity than minimally adjusted models (*I*^2^ = 31.9% *vs*. 90.1%). Importantly, the pooled ORs remained above 1 across these key subgroups, suggesting that the positive association between IBD and subsequent asthma was not driven solely by unadjusted or minimally adjusted studies. Taken together, these findings indicate that inconsistent adjustment for smoking and socioenvironmental factors was likely an important contributor to the high heterogeneity in the OR-based analysis.

For the association between asthma and subsequent IBD, two studies reported HR estimates. Liljendahl et al. (32) and Yun et al. (43) reported HRs of 1.28 (95%CI = 1.06–1.53) and 1.31 (95%CI = 0.53–3.25), respectively. The pooled HR was 1.28 (95%CI = 1.07–1.53), with no observed heterogeneity (*I*^2^ = 0.0%, *p* = 0.961). Two studies reported OR estimates: Fenta et al. (48) and Raj et al. (28) reported ORs of 1.40 (95%CI = 0.62–3.38) and 2.54 (95%CI = 0.78–8.26), respectively. The pooled OR was 1.71 (95%CI = 0.86–3.41), with no observed heterogeneity (*I*^2^ = 0.0%, *p* = 0.422), although the CI crossed the null value. In addition, Krishna et al. (36) reported an RR of 1.44 (95%CI = 1.37–1.51). Overall, the evidence for the IBD-to-asthma direction appeared more consistent than that for the asthma-to-IBD direction.

##### 3.3.3.2 Associations between CD and asthma

**Figure 5:**
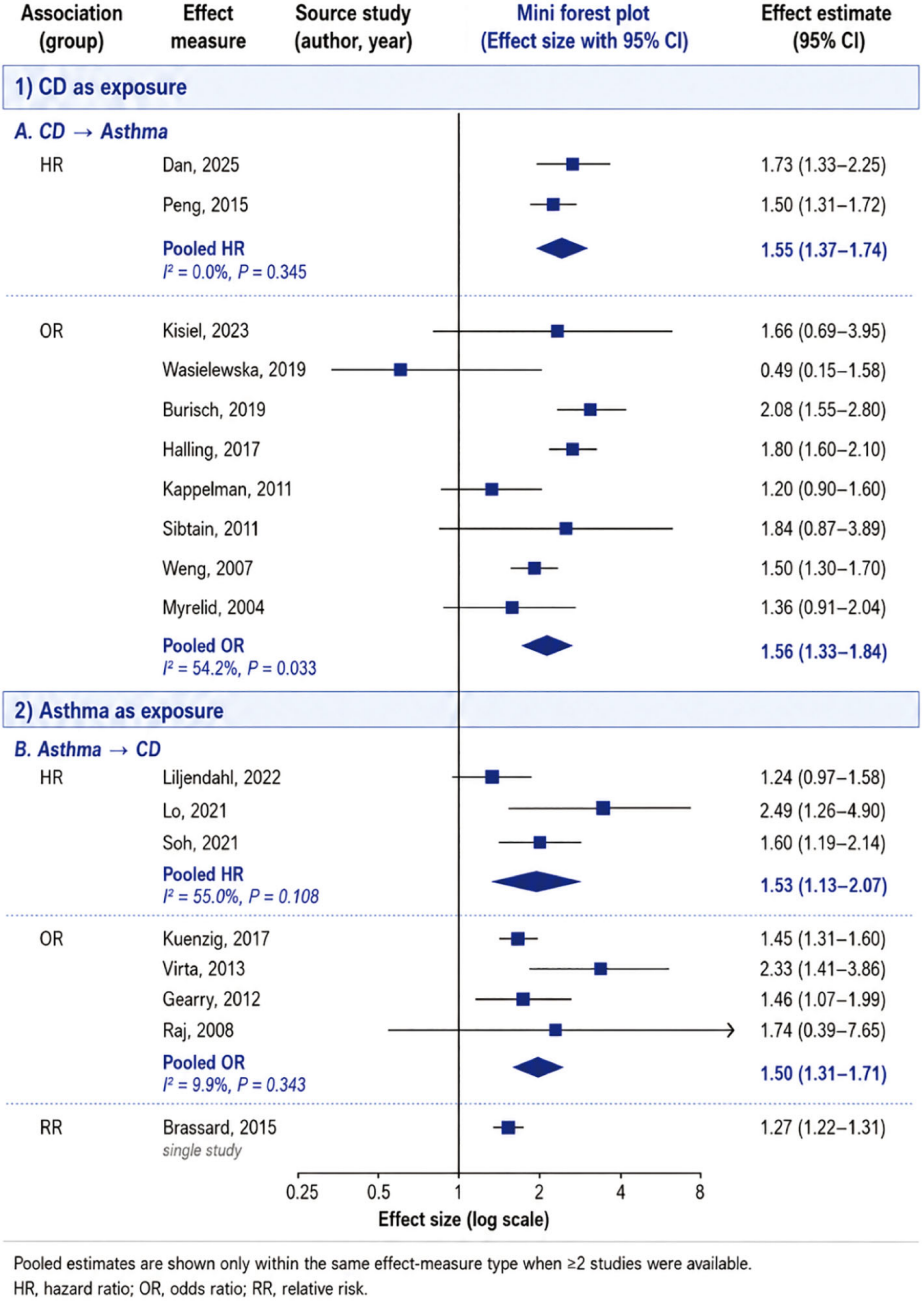
Summary of the bidirectional associations between Crohn’s disease (CD) and asthma, stratified by estimate type.

The bidirectional associations between CD and asthma are summarized in **Figure 5**.

For the association between CD and subsequent asthma, two studies reported HR estimates. Dan et al. (24) reported an HR of 1.73 (95%CI = 1.33–2.25), and Peng et al. (40) reported an HR of 1.50 (95%CI = 1.31–1.72). The pooled HR was 1.55 (95%CI = 1.37–1.74), with no observed heterogeneity (*I*^2^ = 0.0%, *p* = 0.345).

There were eight studies that reported OR estimates for the association between CD and subsequent asthma. The pooled OR was 1.56 (95%CI = 1.33–1.84), with moderate heterogeneity (*I*^2^ = 54.2%, *p* = 0.033). The sensitivity analysis did not identify any individual study that substantially influenced the pooled estimate (**Supplementary Figure 2A**). Subgroup analyses were further conducted according to the analytical method, NOS score, sample size, adjustment for comorbidities, smoking status, atopic diseases, socioenvironmental factors, model complexity, and study design (**Supplementary Table 3**). The results suggested that model complexity may partly explain the observed heterogeneity, as studies with comparable levels of covariate adjustment showed lower within-subgroup heterogeneity. The funnel plot appeared symmetrical (**Supplementary Figure 2B**), and Egger’s test did not suggest significant publication bias (*p* = 0.440).

For the association between asthma and subsequent CD, three studies reported HR estimates. The HRs reported by Liljendahl et al. (32), Lo et al. (33), and Soh et al. (34) were 1.24 (95%CI = 0.97–1.58), 2.49 (95%CI = 1.26–4.90), and 1.60 (95%CI = 1.19–2.14), respectively. The pooled HR was 1.53 (95%CI = 1.13–2.07), with moderate heterogeneity (*I*^2^ = 55.0%, *p* = 0.108). There were four studies that reported OR estimates, yielding a pooled OR of 1.50 (95%CI = 1.31–1.71), with low heterogeneity (*I*^2^ = 9.9%, *p* = 0.343). In addition, Brassard et al. reported an RR of 1.27 (95%CI = 1.22–1.31). Taken together, the HR, OR, and RR analyses consistently supported a positive bidirectional association between CD and asthma.

##### 3.3.3.3 Associations between UC and asthma

**Figure 6:**
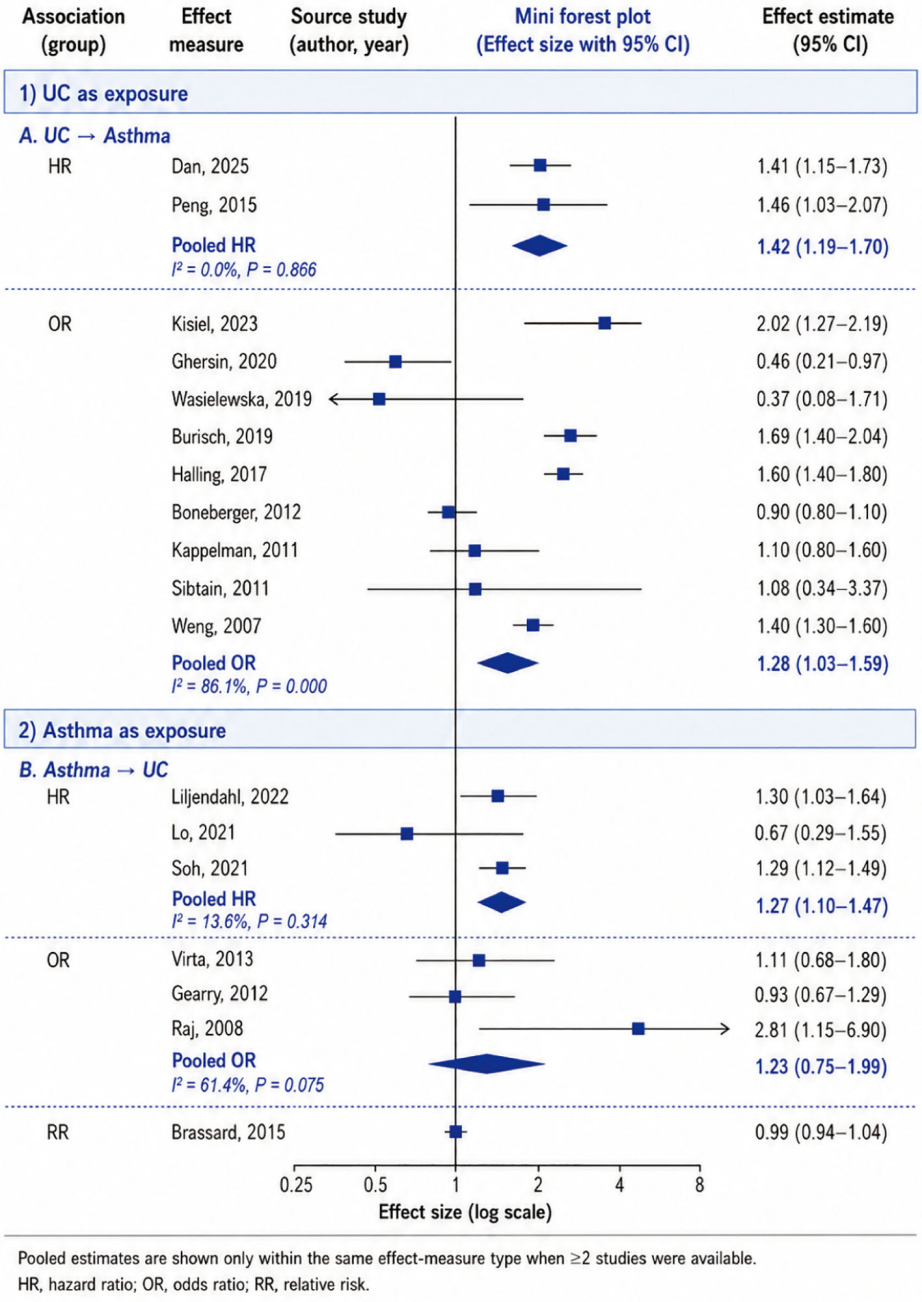
Summary of the bidirectional associations between ulcerative colitis (UC) and asthma, stratified by estimate type.

The bidirectional associations between UC and asthma are summarized in **Figure 6**.

For the association between UC and subsequent asthma, two studies reported HR estimates. Dan et al. (24) reported an HR of 1.41 (95%CI = 1.15–1.73), and Peng et al. (40) reported an HR of 1.46 (95%CI = 1.03–2.07). The pooled HR was 1.42 (95%CI = 1.19–1.70), with no observed heterogeneity (*I*^2^ = 0.0%, *p* = 0.866).

There were nine studies that reported OR estimates for the association between UC and subsequent asthma. The pooled OR was 1.28 (95%CI = 1.03–1.59), indicating a positive association; however, substantial heterogeneity was observed (*I*^2^ = 86.1%, *p* < 0.001). The sensitivity analysis did not identify any individual study with a dominant influence on the pooled estimate (**Supplementary Figure 3A**). The funnel plot did not show obvious publication bias (**Supplementary Figure 3B**), and Egger’s test was also non-significant (*p* = 0.416). Given the high heterogeneity, subgroup analyses were conducted using the same grouping factors as above; however, no specific source of heterogeneity was clearly identified (**Supplementary Table 4**). Unlike the IBD–asthma and CD–asthma analyses, the adjustment-related subgrouping did not clearly resolve the heterogeneity in the UC–asthma analysis. Heterogeneity remained high in both smoking-adjusted and non-smoking-adjusted studies, as well as in studies with minimal and moderate adjustments. Differences by study design and sample size were more apparent: prospective cohort and cross-sectional studies tended to report positive associations, whereas several smaller or retrospective studies reported weaker or inverse estimates. Therefore, the UC–asthma OR-based results should be interpreted as suggestive but less stable than the CD–asthma association. Further inspection of the included studies showed that three studies reported ORs below 1. Among them, Wasielewska et al. (51) and Boneberger et al. (44) had small sample sizes, enrolling only 120 and 226 participants, respectively, and their study populations differed substantially from those of the other included studies, which may have contributed to the unstable estimates. The third study had a large control group, but included only 296 UC cases and seven asthma events, suggesting limited statistical power and potential bias.

For the association between asthma and subsequent UC, three studies reported HR estimates. The HRs reported by Liljendahl et al. (32), Lo et al. (33), and Soh et al. (34) were 1.30 (95%CI = 1.03–1.64), 0.67 (95%CI = 0.29–1.55), and 1.29 (95%CI = 1.12–1.49), respectively. The pooled HR was 1.27 (95%CI = 1.10–1.47), with low heterogeneity (*I*^2^ = 13.6%, *p* = 0.314). There were three studies that reported OR estimates. Virta et al. (41), Gearry et al. (42), and Raj et al. (28) reported ORs of 1.11 (95%CI = 0.68–1.80), 0.93 (95%CI = 0.67–1.29), and 2.81 (95%CI = 1.15–6.90), respectively. The pooled OR was 1.23 (95%CI = 0.75–1.99), with moderate-to-high heterogeneity (*I*^2^ = 61.4%, *p* = 0.075), and the CI crossed the null value. In addition, Brassard et al. (26) reported an RR of 0.99 (95%CI = 0.94–1.04). These findings suggest that the association between UC and subsequent asthma was relatively consistent, whereas the evidence for asthma and subsequent UC was less consistent across different estimate types.

### 3.4 Systematic review of the age profiles and onset age information across the included studies

The age profile and onset age information varied substantially across the included studies. A detailed summary is provided in **Supplementary Table 5**. Overall, the COPD-related evidence was derived mainly from adult or older-adult populations. Dan et al. (24) used UK Biobank data, in which participants were aged 37–73 years at recruitment, with a mean recruitment age of 57.06 ± 8.08 years. The mean age at subsequent COPD diagnosis was 69.32 ± 6.68 years in participants with IBD and was 69.38 ± 6.86 years in those without IBD. The mean age at subsequent asthma diagnosis was also in older adulthood, at 66.07 ± 8.64 and 65.51 ± 8.33 years, respectively. Therefore, this study mainly represents a middle-aged to older-adult population rather than an early-life OLD population.

Other COPD-related studies showed similar age profiles. Lee et al. (25) restricted COPD to adults older than 40 years and reported positive associations in both age strata: for participants aged 40–64 years, the HRs were 1.36 for IBD, 1.31 for UC, and 1.62 for CD; for participants aged ≥65 years, the HRs were 1.46 for IBD, 1.36 for UC, and 1.93 for CD. Ekbom et al. (27) included patients aged ≥40 years at first COPD hospitalization and reported age-stratified estimates for CD after COPD, with HRs of 2.86 for age <60 years, 2.90 for age 60–79 years, and 1.95 for age ≥80 years. Brassard et al. (26) explicitly separated the respiratory disease cohorts by age definition, defining the asthma cohort by respiratory medication use at age ≤40 years and the COPD cohort at age ≥41 years. In that study, the mean age at IBD diagnosis differed markedly between cohorts: in the asthma cohort, the mean ages at CD and UC diagnosis were 26 ± 11 and 31 ± 10 years, respectively; in the COPD cohort, the corresponding ages were 69 ± 11 and 72 ± 11 years. The age-specific incidence rates further supported this distinction: in the asthma cohort, CD incidence was highest at age 20–29 years, 34.3 per 100,000 person-years, while UC incidence was highest at age 30–39 years, 14.9 per 100,000 person-years. In the COPD cohort, CD incidence was highest at age 50–59 years, 35.5 per 100,000 person-years, while UC incidence was highest at age 60–69 years, 24.9 per 100,000 person-years. These findings indicate that the COPD–IBD associations in the current evidence base should be interpreted primarily in the context of adult or older-adult COPD.

Bronchiectasis-related evidence also mainly reflected adult populations. Lee et al. (29) included adults aged ≥20 years and reported a strong age gradient in the IBD cohort. Compared with individuals aged 20–29 years, the multivariable HRs for bronchiectasis were 2.65 for age 30–39 years, 5.27 for age 40–49 years, 7.76 for age 50–59 years, 11.52 for age 60–69 years, and 11.50 for age ≥70 years. Pemmasani et al. (9) used an adult hospitalization-based database, with a mean age of 51.9 ± 19.5 years in both matched IBD and non-IBD groups. Therefore, the available bronchiectasis–IBD evidence is best interpreted as adult bronchiectasis evidence, and current data are insufficient to assess childhood-onset bronchiectasis in relation to IBD.

Asthma-related evidence showed the broadest age spectrum. Several studies explicitly involved childhood, pediatric, or adolescent populations. Liljendahl et al. (32) studied childhood asthma defined by inhaled corticosteroid prescriptions at age 5–7 years and subsequent IBD outcomes. Asthma at age 5–7 years was identified in 18,012 children, accounting for 4.9% of the cohort. Yun et al. (43) also represented a predominantly childhood-onset asthma profile, with a mean asthma onset/index age of 15.1 years and a median age of 5 years (IQR = 1–22 years). Virta et al. (41) investigated pediatric IBD and reported early childhood asthma diagnosis ages: 3.1 years (IQR = 1.9–4.9) in CD, 4.1 years (IQR = 1.9–7.5) in UC, and 3.0 years (IQR = 1.4–5.9) in controls. That study further suggested that asthma diagnosed after age 3 years showed a stronger association with CD than asthma diagnosed before age 3 years. Kappelman et al. (45) also contributed pediatric evidence, including individuals younger than 20 years, with mean ages of approximately 15 years across IBD cases and controls.

In contrast, many of the asthma-related studies were based on adult or mixed-age populations. Peng et al. (40) included adults aged ≥20 years, with a mean age of approximately 46.6 years in the IBD and control groups. Burisch et al. (37) included adult incident IBD cases, with median ages at IBD diagnosis/index of 45.8 years for overall IBD, 43.9 years for CD, and 48.8 years for UC. Halling et al. (38) reported mean ages at study entry of 53 years for IBD, 55 years for UC, and 49 years for CD and also reported mean ages at IBD onset of 42 years for overall IBD, 44 years for UC, and 37 years for CD. Krishna et al. (36) used a large all-age UK primary care database. The asthma cohort had a mean baseline age of 35.61 ± 21.26 years and a mean allergy/asthma diagnosis age of 25.09 ± 21.95 years, indicating that both childhood and adult asthma were represented. Therefore, the asthma–IBD association in the present review should be interpreted as an overall association across mixed age contexts rather than as definitive evidence for a single asthma-onset phenotype.

Some studies provided direct evidence of age-related heterogeneity. Kuenzig et al. (39) reported no significant age effect modification for CD, whereas UC showed evidence of age effect modification, with *p* = 0.0103. The ORs for UC by age at IBD diagnosis/index were 1.49 for age ≤16 years, 1.05 for age 17–40 years, and 1.57 for age >40 years. Jacobsen et al. (10) also included broad IBD age strata, with OLD before IBD/index observed in 11.2% of patients aged ≤16 years, 7.7% of those aged 17–40 years, 11.8% of those aged 41–64 years, and 19.9% of those aged ≥65 years. In the age-stratified follow-up analyses, examples included HR of 1.08 for age ≤16 years and HR of 1.54 for age ≥65 years. These findings suggest that the age profile and the disease onset timing may contribute to heterogeneity across OLD–IBD associations, especially for asthma–UC and bronchiectasis-related outcomes. However, because the age strata were not harmonized across studies and the majority of the studies did not report comparable age-at-onset-specific estimates, the available evidence supports systematic narrative synthesis rather than formal age-stratified pooling.

## 4 Discussion

Through systematic review and meta-analysis, the present study incorporated COPD, asthma, and bronchiectasis into a unified framework and evaluated their bidirectional associations with IBD and its subtypes using estimate-stratified analyses. The main findings were as follows. First, IBD is associated with an increased risk of subsequent COPD, and COPD is generally associated with increased risks of subsequent CD and UC, although some COPD-to-IBD subtype analyses showed substantial heterogeneity. Second, available evidence suggests a positive association between IBD and bronchiectasis; however, the pooled estimate for IBD and subsequent bronchiectasis was imprecise and should be interpreted with caution. Third, the association between asthma and IBD was relatively consistent, especially for the IBD-to-asthma and CD–asthma associations. Fourth, the association between asthma and UC was less consistent across different estimate types, particularly in the asthma-to-UC direction.

The findings of the present study are broadly consistent with previous systematic reviews focusing on individual OLD–IBD associations while substantially expanding the evidence base in both study number and sample size (18, 19). For the COPD–IBD association, Labarca et al. included four observational studies and reported a pooled HR of 2.02 (95%CI = 1.56–2.63) for new-onset IBD in patients with COPD. Their GRADE summary table reported 660,463 participants across the four studies (18). In comparison, our COPD-related analysis included six studies, adding two more studies, and the total sample size increased to 8,818,786 participants, representing an increase of 8,158,323 participants and an approximately 13.4-fold expansion of the evidence base. Consistent with Labarca et al., our estimate-stratified analyses showed positive associations between COPD and subsequent IBD-related outcomes, particularly CD, with a pooled HR of 2.16 (95%CI = 1.36–3.44) for COPD and subsequent CD and a pooled HR of 1.55 (95%CI = 1.12–2.14) for COPD and subsequent UC.

Similarly, Kuenzig et al. included 18 studies in their systematic review of asthma and IBD, including 15 studies on CD and 15 studies on UC, and reported significant associations of asthma with both CD (pooled RR = 1.30, 95%CI = 1.16–1.47) and UC (pooled RR = 1.34, 95%CI = 1.24–1.44) (19). In the present study, the asthma-related analysis included 27 studies, adding nine studies compared with Kuenzig et al. Among the studies with available sample size information, the total sample size reached 8,426,609 participants. Our updated estimate-stratified analyses further supported the asthma–IBD association. For overall IBD and subsequent asthma, the pooled HR and OR were 1.56 (95%CI = 1.46–1.67) and 1.51 (95%CI = 1.29–1.77), respectively. For CD and subsequent asthma, the pooled HR and OR were 1.55 (95%CI = 1.37–1.74) and 1.56 (95%CI = 1.33–1.84), respectively. For asthma and subsequent CD, the pooled HR and OR were 1.53 (95%CI = 1.13–2.07) and 1.50 (95%CI = 1.31–1.71), respectively. These results indicate a relatively consistent bidirectional association between CD and asthma. In contrast, the association between asthma and UC appeared less consistent across estimate types. Although UC was associated with subsequent asthma in both HR- and OR-based analyses, with pooled estimates of 1.42 (95%CI = 1.19–1.70) and 1.28 (95%CI = 1.03–1.59), respectively, the pooled OR for asthma and subsequent UC was 1.23 (95%CI = 0.75–1.99), crossing the null value.

Importantly, because the present analysis separated the HR, RR, and OR estimates rather than converting all estimate types into a single metric, direct numerical comparisons with previous pooled RR or OR estimates should be made cautiously. Nevertheless, compared with previous systematic reviews, the present study not only added more recent large-scale population-based studies but also extended the analytical framework by incorporating COPD, asthma, and bronchiectasis simultaneously, evaluating bidirectionality, disease subtype specificity, estimate type differences, and sources of heterogeneity.

Substantial heterogeneity was observed in several pooled analyses, particularly for COPD and subsequent CD, IBD and subsequent bronchiectasis, IBD and subsequent asthma based on OR estimates, and UC and subsequent asthma based on OR estimates. This heterogeneity may be partly explained by differences in the study design, diagnostic definitions, population characteristics, and covariate adjustment. For example, COPD was defined using different approaches across studies, including ICD codes, medication records, clinical criteria, and prescription-based algorithms, such as the definition used by Brassard et al. (26). Similarly, bronchiectasis was identified using either administrative codes or clinical and CT-based criteria, while some studies, such as that by Raj et al. (28), had relatively small sample sizes and did not report multivariable adjustment.

In the asthma-related analyses, the larger number of included studies allowed further exploration of heterogeneity. Subgroup analyses showed that heterogeneity was reduced in studies with more adequate covariate adjustment, particularly those adjusted for smoking or socioenvironmental factors, whereas minimally adjusted studies retained substantial heterogeneity. Differences in the asthma and IBD definitions, including the ICD codes, medication records, self-report, allergological diagnosis, and clinical criteria, may have also contributed to the between-study variability. For the UC–asthma association, several small studies or studies with limited asthma events, such as those by Wasielewska et al. (51) and Boneberger et al. (44), reported estimates below 1, which may partly explain the instability of the pooled results. Overall, the observed heterogeneity does not negate the association between OLD and IBD, but highlights the complexity of these relationships across different populations and methodological settings.

The subgroup analyses also added clinically relevant information beyond the pooled estimates. In the OR-based asthma analyses, the reduction in heterogeneity after stratification by smoking adjustment, socioenvironmental adjustment, and model complexity suggests that differences in confounder control were a major source of between-study variability (**Supplementary Tables 2**-**4**). This is biologically and epidemiologically plausible because smoking, socioeconomic status, environmental exposures, comorbid allergic diseases, and healthcare access may influence both asthma diagnosis and IBD risk (1, 2, 5, 14). Importantly, the positive association between IBD and subsequent asthma, and particularly between CD and subsequent asthma, persisted in the multivariable-adjusted strata, supporting the robustness of these associations (**Supplementary Tables 2**, **3**). In contrast, the UC–asthma association was less stable across the adjustment and design strata, which is consistent with the weaker and more heterogeneous estimates observed in the main analysis (**Supplementary Table 4**).

The age profile synthesis provides important clinical context for interpreting the present findings. COPD and bronchiectasis were predominantly represented by adult or older-adult cohorts in the available literature, whereas asthma-related studies included childhood-onset, pediatric, adolescent, adult, and mixed-age populations. This distinction is clinically important because the mechanisms linking OLD and IBD may differ by life stage. Associations involving COPD may reflect adult or older-adult respiratory disease, shared environmental exposures such as smoking, systemic inflammation, comorbidity burden, and healthcare surveillance. The age-specific incidence rates reported by Brassard et al. (26) further support this interpretation, as the IBD diagnoses after asthma-like disease were concentrated in the younger age groups, whereas the IBD diagnoses after COPD were concentrated in the older age groups. Similarly, the marked age gradient in bronchiectasis risk reported by Lee et al. (29) suggests that age itself is a major determinant of bronchiectasis occurrence in IBD populations.

In contrast, asthma–IBD associations may involve both early-life immune dysregulation and later-life inflammatory or environmental pathways. Pediatric studies such as those by Liljendahl et al. (32), Yun et al. (43), Virta et al. (41), and Kappelman et al. (45) support the relevance of childhood or adolescent asthma/IBD contexts, whereas large adult or mixed age studies such as those by Dan et al. (24), Peng et al. (40), Burisch et al. (37), Halling et al. (38), Krishna et al. (36), and Kuenzig et al. (39) indicate that asthma–IBD associations are not limited to early life. Importantly, Kuenzig et al. (39) suggested age-related heterogeneity for UC but not for CD, indicating that age may influence disease subtype-specific associations. Therefore, the present results should not be interpreted as evidence that all OLD subtypes share the same age-related relationship with IBD. Rather, they support subtype-specific and age context-specific interpretation.

Overall, we observed generally positive associations between OLD and IBD, although the strength and stability of these associations varied across disease combinations and estimate types. The underlying pathophysiological mechanisms are likely complex and disease-specific.

First, the mechanisms underlying the association between COPD and IBD may involve multilevel interactions, which may help explain why the association appeared stronger for CD than for UC in our estimate-stratified analyses. In the HR-based analyses, COPD was associated with subsequent CD, with a pooled HR of 2.16, whereas the pooled HR for COPD and subsequent UC was 1.55. Firstly, smoking, as a key environmental factor, can disrupt intestinal epithelial tight junctions, induce intestinal flora imbalance, and activate pro-inflammatory pathways such as NF-κB in the intestinal mucosa. This pathway preferentially exacerbates Th17-type intestinal inflammation characteristic of CD, jointly promoting the occurrence of IBD (52, 53). Secondly, microbiome disturbance serves as an important bridge: metabolites derived from the intestinal flora (such as short-chain fatty acids) can affect pulmonary immunity through circulation, and pulmonary inflammation can also reversely regulate the composition and function of the intestinal flora through immune cell circulation (54, 55). Thirdly, both diseases share immune dysregulation characterized by sustained activation of pro-inflammatory factor networks including the IL-1β, IL-6, TNF-α, and Th17 pathway. The systemic inflammatory profile of CD overlaps significantly more with COPD than UC (56–58), which is more inclined to local intestinal epithelial damage, thus showing a stronger association. Genetic studies have also identified common susceptible loci (such as IL8 and TNF, among others), suggesting that some patients have an inherent genetic background leading to mucosal immune homeostasis imbalance. Such genetic susceptibility exerts a more significant impact on lung and ileal mucosal barriers, further elevating the risk of CD in patients with COPD (17, 59).

Second, the association between asthma and IBD may be mediated by distinct immune and microbiome-related mechanisms, which may explain the bidirectional but subtype-specific patterns observed in this study. The association was relatively consistent between CD and asthma: CD was associated with subsequent asthma in both HR- and OR-based analyses, and asthma was also associated with subsequent CD. In contrast, the association between asthma and UC was less consistent across estimate types, particularly in the asthma-to-UC direction. Traditionally, asthma is considered a Th2-predominant disease; in IBD, CD is dominated by Th1/Th17 responses (60), while UC is associated with atypical Th2 responses and other pathways (61). However, emerging evidence indicates that these diseases with seemingly distinct mechanisms share some common immune regulatory disorders. Abnormal regulatory T-cell (Treg) function may affect both airway hyperresponsiveness and intestinal immune tolerance (61, 62). Genome-wide association studies (GWAS) have identified multiple susceptible loci for IBD (63), among which genes such as *SMAD3* and *TNFSF15* have also been found to play roles in other immune diseases (e.g., asthma). The stronger systemic inflammatory feature of CD can more significantly affect airway immunity through circulation and aggravate airway hyperresponsiveness, whereas UC inflammation is relatively localized (56–58), leading to a weaker association with asthma. On the other hand, systemic immune priming mediated by IBD is more likely to lower the threshold of allergic airway responses, resulting in a slightly stronger association of IBD with subsequent asthma than that of asthma with subsequent IBD, forming asymmetry in bidirectional association strength. Notably, the mediating role of the microbiome has attracted increasing attention, and the “gut–lung axis” modulates systemic immune responses through microbiome–immune system interactions (64). Specifically, respiratory flora imbalance in asthma patients may affect intestinal flora composition through circulating immune cells, and altered intestinal flora may remotely regulate pulmonary immune responses, forming a bidirectional regulatory network (65, 66).

Finally, the association between bronchiectasis and IBD may have unique features in addition to immune and microbiome dysregulation. Although the available studies generally suggest a positive association, the pooled estimate for IBD and subsequent bronchiectasis was imprecise and should be interpreted with caution due to the limited number of studies and substantial heterogeneity. Persistent chronic pulmonary infection and inflammation may lead to a systemic inflammatory state, which in turn impairs intestinal barrier function. Neutrophils play a central role in the pathology of bronchiectasis, and activated neutrophils release proteases and reactive oxygen species, which may enter the circulatory system and damage intestinal mucosal integrity (67).

Notably, drug treatments may also partially explain these associations. Immunosuppressants used by patients with IBD may increase the risk of respiratory infections, thereby promoting bronchiectasis development. Conversely, some medications used for asthma treatment may modulate systemic immune responses, influencing intestinal inflammation (68).

In summary, the association between OLD and IBD likely involves multiple mechanisms, including genetic susceptibility, environmental factors (especially smoking), immune system dysregulation, microbiome imbalances, and the potential role of pharmacological treatments. These mechanisms likely vary in importance across different OLD–IBD combinations, which may explain the observed differences in risk in this study.

The findings of this study hold significant clinical relevance. For gastroenterologists, heightened awareness of coexisting OLD, particularly in patients with CD, is essential when diagnosing and managing IBD. Proactively inquiring about chronic respiratory symptoms—such as cough, sputum production, and shortness of breath—and considering pulmonary function screening may facilitate early identification and intervention, ultimately improving patient outcomes. Similarly, pulmonologists treating patients with COPD, refractory asthma, or idiopathic bronchiectasis should be alert to the presence of unexplained abdominal pain, diarrhea, or bloody stools, which could offer early diagnostic clues for IBD.

The present study has several limitations. First, the conclusions of this meta-analysis are dependent on the quality of the included observational studies. Although the majority of the studies were of high quality according to the NOS scores, residual confounding remains unavoidable. Second, moderate to substantial heterogeneity was observed in several analyses. Although subgroup and sensitivity analyses were performed, the sources of heterogeneity could not be fully explained in all comparisons, particularly for bronchiectasis-related analyses and UC–asthma associations. Third, information on the age at onset was incompletely and inconsistently reported across the included studies. Although we systematically extracted age eligibility, baseline age, age at diagnosis or index date, and age-stratified findings when available, the included studies used different age categories and rarely reported comparable age-at-onset-specific association estimates. For example, some studies defined asthma in early childhood, some used all-age or adult asthma cohorts, and COPD studies generally represented middle-aged or older adults. Therefore, formal age-stratified meta-analysis was not feasible. The age-related findings in this review should be interpreted as a systematic narrative synthesis rather than quantitative proof of age-specific causal pathways. Fourth, different studies reported different types of association estimates, including HRs, ORs, and RRs. To avoid inappropriate conversion across estimate types, we analyzed these estimates separately. However, this approach reduced the number of studies available for each pooled analysis and limited the statistical power of some comparisons. Therefore, the results based on only one or two studies should be interpreted with caution. Fifth, although four studies investigated bronchiectasis-related associations, only a small number of studies were available for each direction and estimate type. This limited the stability of the pooled estimates and precluded robust subgroup analyses. Finally, the included studies varied in diagnostic criteria, population characteristics, follow-up duration, and covariate adjustment, which may have contributed to variability in the observed associations.

## 5 Conclusion

In conclusion, this systematic review and meta-analysis supports positive associations between OLD and IBD, particularly for COPD with the IBD subtypes and for asthma with IBD, especially CD. These associations should be interpreted according to the OLD subtype and the age context, as the evidence for COPD and bronchiectasis mainly reflects adult or older-adult populations, whereas the evidence for asthma includes mixed age groups. Clinicians should be attentive to respiratory comorbidities in patients with IBD, and appropriate respiratory symptom assessment or screening may help early identification and management.

## Data Availability

The original contributions presented in the study are included in the article/**Supplementary Material**. Further inquiries can be directed to the corresponding authors.
