# Supplementary material for: Association between OLD and IBD: a systematic review and meta-analysis
Source: Front Immunol. 2026 Jun 30;17:1726732. doi: 10.3389/fimmu.2026.1726732 (PMC13364986; doi:10.3389/fimmu.2026.1726732)
Supplement: Supplementary file 1 [file Table1.docx]

Supplementary Material

Contents

[1 Supplementary Tables 2](#_Toc231164372)

[Supplementary Table 1. The details of search strategies. 2](#_Toc231164373)

[Supplementary Table 2. Subgroup analyses of OR estimates for the association between IBD and the risk of asthma. 5](#_Toc231164374)

[Supplementary Table 3. Subgroup analyses of OR estimates for the association between Crohn’s disease and the risk of asthma. 6](#_Toc231164375)

[Supplementary Table 4. Subgroup analyses of OR estimates for the association between ulcerative colitis and the risk of asthma. 7](#_Toc231164376)

[Supplementary Table 5. Simplified age profiles and key age-stratified findings of included studies. 8](#_Toc231164377)

[2 Supplementary Figures 14](#_Toc231164378)

[Supplementary Figure 1. Sensitivity analysis and funnel plot for the association between IBD and subsequent asthma based on OR estimates. 14](#_Toc231164379)

[Supplementary Figure 2. Sensitivity analysis and funnel plot for the association between Crohn’s disease and subsequent asthma based on OR estimates. 15](#_Toc231164380)

[Supplementary Figure 3. Sensitivity analysis and funnel plot for the association between ulcerative colitis and subsequent asthma based on OR estimates. 16](#_Toc231164381)

# Supplementary Tables

Supplementary Table 1. The details of search strategies.

| Database | Search step | Search strategy | Search result |
| --- | --- | --- | --- |
| PubMed |  |  |  |
|  | #1 | ((((((Lung Disease, Obstructive) OR (Obstructive Lung Disease)) OR (Obstructive Lung Diseases)) OR (Obstructive Pulmonary Diseases)) OR (Obstructive Pulmonary Disease))OR (Pulmonary Disease, Obstructive)) OR (Pulmonary Diseases, Obstructive) | 285,766 |
|  |  |  |  |
|  | #2 | ((((((((Chronic Obstructive Lung Disease) OR (Chronic Obstructive Pulmonary Diseases)) OR (COPD)) OR (Chronic Obstructive Airway Disease)) OR (Chronic Obstructive Pulmonary Disease)) OR (Airflow Obstruction, Chronic)) OR (Airflow Obstructions, Chronic)) OR (Chronic Airflow Obstructions)) OR (Chronic Airflow Obstruction) | 123,276 |
|  |  |  |  |
|  | #3 | ((Asthmas) OR (Asthma, Bronchial)) OR (Bronchial Asthma) | 233,057 |
|  |  |  |  |
|  | #4 | ((((((((((((Bronchiectases) OR (Cylindrical Bronchiectasis)) OR (Bronchiectasis, Cylindrical)) OR (Cylindrical Bronchiectases)) OR (Varicose Bronchiectasis)) OR (Bronchiectasis, Varicose)) OR (Varicose Bronchiectases)) OR (Saccular Bronchiectasis)) OR (Bronchiectasis, Saccular)) OR (Saccular Bronchiectases)) OR (Cystic Bronchiectasis)) OR (Bronchiectasis, Cystic)) OR (Cystic Bronchiectases) | 17,401 |
|  |  |  |  |
|  | #5 | (((((((((Fibrosis, Cystic) OR (Mucoviscidosis)) OR (Pancreatic Cystic Fibrosis)) OR (Cystic Fibrosis, Pancreatic)) OR (Cystic Fibrosis of Pancreas)) OR (Fibrocystic Disease of Pancreas)) OR (Pancreas Fibrocystic Disease)) OR (Pancreas Fibrocystic Diseases)) OR (Pulmonary Cystic Fibrosis)) OR (Cystic Fibrosis, Pulmonary) | 66,102 |
|  |  |  |  |
|  | #6 | #1 OR #2 OR #3 OR #4 OR #5 | 440,149 |
|  |  |  |  |
|  | #7 | (((((((((((((((((((((Inflammatory Bowel Disease) OR (Bowel Diseases, Inflammatory)) OR (Crohn's Disease)) OR (Crohns Disease)) OR (Crohn's Enteritis)) OR (Inflammatory Bowel Disease 1)) OR (Regional Enteritis)) OR (Ileocolitis)) OR (Ileitis, Terminal)) OR (Terminal Ileitis)) OR (Ileitis, Regional)) OR (Regional Ileitides)) OR (Regional Ileitis)) OR (Enteritis, Granulomatous)) OR (Granulomatous Enteritis)) OR (Enteritis, Regional)) OR (Colitis, Granulomatous)) OR (Granulomatous Colitis)) OR (Colitis Gravis)) OR (Idiopathic Proctocolitis)) OR (Inflammatory Bowel Disease, Ulcerative Colitis Type)) OR (Ulcerative Colitis) | 161,676 |
|  |  |  |  |
|  | #8 | #6 AND #7 | 2,834 |
|  |  |  |  |
|  | #9 | review[Publication Type] | 3,657,654 |
|  |  |  |  |
|  | #10 | #8 NOT #9 | 1,679 |
|  |  |  |  |
| Embase |  |  |  |
|  | #1 | 'chronic obstructive lung disease'/exp OR 'chronic obstructive lung disease' OR 'obstructive lung disease'/exp OR 'obstructive lung disease' OR 'asthma'/exp OR 'asthma' OR 'bronchiectasis'/exp OR 'bronchiectasis' OR 'cystic fibrosis'/exp OR 'cystic fibrosis' | 723,387 |
|  |  |  |  |
|  | #2 | 'inflammatory bowel disease'/exp OR 'inflammatory bowel disease' OR 'crohn disease'/exp OR 'crohn disease' OR 'ulcerative colitis'/exp OR 'ulcerative colitis' | 270,464 |
|  |  |  |  |
|  | #3 | [article]/lim | 31,579,305 |
|  |  |  |  |
|  | #4 | #1 AND #2 AND #3 | 3,326 |
|  |  |  |  |
|  | #5 | review:ti | 974,817 |
|  |  |  |  |
|  | #6 | #4 NOT #5 | 3,213 |
|  |  |  |  |
| Cochrane Library |  |  |  |
|  | #1 | MeSH descriptor: [Pulmonary Disease, Chronic Obstructive] explode all trees | 8,032 |
|  |  |  |  |
|  | #2 | MeSH descriptor: [Cystic Fibrosis] explode all trees | 2,314 |
|  |  |  |  |
|  | #3 | MeSH descriptor: [Bronchiectasis] explode all trees | 492 |
|  |  |  |  |
|  | #4 | MeSH descriptor: [Asthma] explode all trees | 14,654 |
|  |  |  |  |
|  | #5 | #1 OR #2 OR #3 OR #4 | 25,064 |
|  |  |  |  |
|  | #6 | MeSH descriptor: [Inflammatory Bowel Diseases] explode all trees | 5,035 |
|  |  |  |  |
|  | #7 | MeSH descriptor: [Crohn Disease] explode all trees | 2,340 |
|  |  |  |  |
|  | #8 | MeSH descriptor: [Colitis, Ulcerative] explode all trees | 2,324 |
|  |  |  |  |
|  | #9 | #6 OR #7 OR #8 | 5,035 |
|  |  |  |  |
|  | #10 | #5 AND #9 | 9 |
|  |  |  |  |

Supplementary Table 2. Subgroup analyses of OR estimates for the association between IBD and the risk of asthma.

| Variables | No. of studies | OR (95%CI) | Heterogeneity | | Model |
| --- | --- | --- | --- | --- | --- |
|  |  |  | I^2^ (%) | P |  |
| **Sample size** |  |  |  |  |  |
| > 1000 | 6 | 1.61 (1.43 - 1.81) | 88.6 | 0.000 | Random |
| < 1000 | 1 | 1.32 (0.75 - 2.31) | - | - | Fixed |
| **Smoking adj** |  |  |  |  |  |
| Yes | 2 | 1.51 (1.41 - 1.61) | 31.9 | 0.226 | Fixed |
| No | 5 | 1.60 (1.36 - 1.89) | 90.1 | 0.000 | Random |
| **Socioenvironmental factors adj** |  |  |  |  |  |
| Yes | 3 | 1.71 (1.58 - 1.84) | 0.0 | 0.816 | Fixed |
| No | 4 | 1.52 (1.33 - 1.73) | 86.8 | 0.000 | Random |
| **Model complexity** |  |  |  |  |  |
| Minimal | 5 | 1.60 (1.36 - 1.89) | 90.1 | 0.000 | Random |
| Moderate | 2 | 1.51 (1.41 - 1.61) | 31.9 | 0.226 | Fixed |
| **Study designs** |  |  |  |  |  |
| Prospective cohort | 3 | 1.53 (1.16 - 2.01) | 88.7 | 0.000 | Random |
| Cross-sectional study | 2 | 1.71 (1.57 - 1.86) | 0.0 | 0.530 | Fixed |
| Case control study | 2 | 1.52 (1.43 - 1.62) | 21.0 | 0.260 | Fixed |
| IBD: Inflammatory Bowel Disease. Unadjusted: no covariates; Minimal: age and/or sex only; Moderate: age and sex plus ≤3 additional covariates; Full: age and sex plus >3 additional covariates. | | | | | |

Supplementary Table 3. Subgroup analyses of OR estimates for the association between Crohn’s disease and the risk of asthma.

| Variables | No. of studies | OR (95%CI) | Heterogeneity | | Model |
| --- | --- | --- | --- | --- | --- |
|  |  |  | I^2^ (%) | P |  |
| **Analysis** |  |  |  |  |  |
| M | 7 | 1.60 (1.39 - 1.84) | 47.1 | 0.079 | Random |
| U | 1 | 0.49 (0.15 - 1.59) | - | - | Fixed |
| **NOS** |  |  |  |  |  |
| ≥ 7 | 7 | 1.60 (1.39 - 1.84) | 47.1 | 0.079 | Random |
| < 7 | 1 | 0.49 (0.15 - 1.59) | - | - | Fixed |
| **Sample size** |  |  |  |  |  |
| > 1000 | 6 | 1.59 (1.37 - 1.85) | 55.4 | 0.047 | Random |
| < 1000 | 2 | 1.03 (0.29 - 3.71) | 71.1 | 0.063 | Random |
| **Smoking adj** |  |  |  |  |  |
| Yes | 2 | 1.50 (1.32 - 1.72) | 0.0 | 0.822 | Fixed |
| No | 6 | 1.55 (1.22 - 1.97) | 63.2 | 0.018 | Random |
| **Atopy adj** |  |  |  |  |  |
| Yes | 1 | 1.36 (0.91 - 2.04) | - | - | Fixed |
| No | 7 | 1.59 (1.33 - 1.89) | 58.9 | 0.024 | Random |
| **Socioenvironmental factors adj** |  |  |  |  |  |
| Yes | 4 | 1.50 (1.16 - 1.92) | 58.1 | 0.067 | Random |
| No | 4 | 1.61 (1.16 - 2.21) | 61.9 | 0.049 | Random |
| **Model complexity** |  |  |  |  |  |
| Unadjusted | 1 | 0.49 (0.15 - 1.58) | - | - | Fixed |
| Minimal | 3 | 1.85 (1.63 - 2.09) | 0.0 | 0.684 | Fixed |
| Moderate | 4 | 1.44 (1.28 - 1.61) | 0.0 | 0.557 | Fixed |
| **Study designs** |  |  |  |  |  |
| Prospective cohort | 2 | 2.05 (1.55 - 2.69) | 0.0 | 0.765 | Fixed |
| Retrospective cohort | 1 | 0.49 (0.15 - 1.59) | - | - | Random |
| Cross-sectional study | 5 | 1.52 (1.30 - 1.78) | 51.0 | 0.086 | Random |
| M: multivariate-adjusted; U: unadjusted. Unadjusted: no covariates; Minimal: age and/or sex only; Moderate: age and sex plus ≤3 additional covariates; Full: age and sex plus >3 additional covariates. | | | | | |

Supplementary Table 4. Subgroup analyses of OR estimates for the association between ulcerative colitis and the risk of asthma.

| Variables | No. of studies | OR (95%CI) | Heterogeneity | | Model |
| --- | --- | --- | --- | --- | --- |
|  |  |  | I^2^ (%) | P |  |
| **Analysis** |  |  |  |  |  |
| M | 8 | 1.31 (1.20 - 1.47) | 87.2 | 0.000 | Random |
| U | 1 | 0.37 (0.08 - 1.71) | - | - | Fixed |
| **Sample size** |  |  |  |  |  |
| > 1000 | 6 | 1.47 (1.24 - 1.75) | 76.3 | 0.001 | Random |
| < 1000 | 3 | 0.89 (0.76 - 1.05) | 0.0 | 0.495 | Fixed |
| **Comorbidity adj** |  |  |  |  |  |
| Yes | 1 | 0.46 (0.21 - 0.99) | - | - | Fixed |
| No | 8 | 1.36 (1.11 - 1.68) | 85.9 | 0.000 | Random |
| **Socioenvironmental factors adj** |  |  |  |  |  |
| Yes | 3 | 1.56 (1.20 - 2.04) | 72.6 | 0.026 | Random |
| No | 6 | 1.09 (0.79 - 1.49) | 87.4 | 0.000 | Random |
| **Smoking adj** |  |  |  |  |  |
| Yes | 2 | 1.64 (1.15 - 2.35) | 83.5 | 0.014 | Random |
| No | 7 | 1.10 (0.81 - 1.51) | 87.8 | 0.000 | Random |
| **Model complexity** |  |  |  |  |  |
| Unadjusted | 1 | 0.37 (0.08 - 1.70) | - | - | Fixed |
| Minimal | 4 | 1.32 (0.92 - 1.91) | 92.0 | 0.000 | Random |
| Moderate | 4 | 1.27 (0.90 - 1.79) | 82.3 | 0.001 | Random |
| **Study designs** |  |  |  |  |  |
| Prospective cohort | 2 | 1.67 (1.39 - 2.01) | 0.0 | 0.450 | Fixed |
| Retrospective cohort | 2 | 0.44 (0.22 - 0.87) | 0.0 | 0.798 | Fixed |
| Cross-sectional study | 2 | 1.74 (1.40 - 2.17) | 56.9 | 0.128 | Random |
| Case-Control study | 3 | 1.12 (0.81 - 1.56) | 90.5 | 0.000 | Random |
| M: multivariate-adjusted; U: unadjusted. Unadjusted: no covariates; Minimal: age and/or sex only; Moderate: age and sex plus ≤3 additional covariates; Full: age and sex plus >3 additional covariates. | | | | | |

Supplementary Table 5. Simplified age profiles and key age-stratified findings of included studies.

| **Study** | **OLD subtype** | **Population** | **Age profile / key findings** | **Interpretation** |
| --- | --- | --- | --- | --- |
| Dan,2025 | COPD | UK Biobank; 430,414 participants after exclusions | UK Biobank recruitment; source article states participants were aged 37–73 years; mean age at recruitment 57.06 ± 8.08 years overall; CD, 56.94 ± 8.19; UC, 58.02 ± 7.87. Stratification by age at recruitment was performed; the article reported no evidence of interaction by age. | Represents a middle-aged to older adult cohort; findings are most relevant to adult/older-adult respiratory disease rather than early-life obstructive lung disease. |
| Jacobsen,2024 | COPD | Danish nationwide registries; incident IBD cohort and age-, sex-, calendar-period-, and municipality-matched reference cohort | Overall mean age at IBD/index: 43.1 ± 19.7 years. Age groups explicitly reported: ≤16, 17–40, 41–64, ≥65 years. OLD before IBD/index in IBD cohort: ≤16, 182/1620 (11.2%); 17–40, 794/10,264 (7.7%); 41–64, 938/7970 (11.8%); ≥65, 871/4384 (19.9%). Age-stratified HRs after IBD were reported; examples include ≤16 HR 1.08 (0.87–1.33) and ≥65 HR 1.54 (1.41–1.67). | Important source for age profile: both pediatric/young adult and older-adult IBD groups were represented; age strata should be described narratively rather than pooled. |
| Lee,2019 | COPD | South Korean National Health Insurance Service database; COPD cohort and matched controls | COPD was defined in adults >40 years. COPD cohort n = 1,303,021 and controls n = 6,515,105; mean age 57.1 ± 10.72 years in both groups. Age groups: 40–64 years 77.1%; ≥65 years 22.9%. Age-stratified HRs: age 40–64 years: IBD 1.36 (1.21–1.52), UC 1.31 (1.15–1.48), CD 1.62 (1.25-2.08); age ≥65 years: IBD 1.46 (1.19-1.79), UC 1.36 (1.07-1.71), CD 1.93 (1.23-2.96). | Strong age-relevant COPD study; findings apply to adult COPD and suggest association in both 40–64 and ≥65 strata. |
| Brassard,2015 | COPD | Quebec administrative databases; asthma cohort, COPD cohort, and age-, sex-, region-, and index-year-matched general-population cohorts | Asthma cohort: respiratory medication algorithm with cohort entry at age ≤40 years. COPD cohort: third respiratory medication prescription at age ≥41 years; subjects with asthma codes excluded from COPD cohort. Age-specific incidence rates were reported. Asthma cohort: CD incidence highest at age 20–29 years (34.3/100,000 person-years); UC highest at age 30–39 years (14.9/100,000 person-years). COPD cohort: CD highest at 50–59 years (35.5/100,000 person-years); UC highest at 60–69 years (24.9/100,000 person-years). | Provides explicit age-based distinction between asthma-like disease and COPD cohorts and reports age-specific IBD incidence rates. |
| Ekbom,2008 | COPD | Swedish Inpatient Register; patients aged ≥40 years with COPD and matched controls; also first-degree relatives | Patients with COPD were aged ≥40 years at first hospital discharge with COPD; analyses stratified by age at first discharge: <60, 60–79, and ≥80 years. Age-stratified estimates were reported. For CD after COPD: <60 HR 2.86 (2.10-3.94), 60–79 HR 2.90 (2.36-3.58), ≥80 HR 1.95 (1.32-2.92). UC estimates also stratified by <60, 60–79, and ≥80 years. | Adult/older-adult COPD evidence; useful for age-stratified narrative synthesis. |
| Raj,2008 | COPD / bronchiectasis / asthma | Respiratory clinic-based cohort with obstructive airway disease, including COPD, asthma, and bronchiectasis | Respiratory-clinic population; no age-matched population comparison. Among IBD cases with respiratory disease, mean age was approximately 61 ± 3 years for UC and 60 ± 4 years for CD. No age-stratified estimates. Authors noted comparisons with the general population were not adjusted for age/smoking. | Exploratory adult clinical sample; large ORs should be interpreted cautiously because of small numbers and limited age adjustment. |
| Lee,2024 | Bronchiectasis | South Korean NHIS database; adults with IBD and matched controls | Adults aged ≥20 years. IBD cohort n = 6,513 and controls n = 26,052. Age categories: 20–29, 30–39, 40–49, 50–59, 60–69, ≥70 years. Risk of bronchiectasis increased with age in the IBD cohort; compared with 20–29 years, multivariable HRs were 2.65 (30–39), 5.27 (40–49), 7.76 (50–59), 11.52 (60–69), and 11.50 (≥70). | Adult bronchiectasis evidence; age is a strong risk factor for bronchiectasis and should be described narratively. |
| Pemmasani,2022 | Bronchiectasis | US National Readmissions Database; matched IBD and non-IBD hospitalizations | IBD and matched non-IBD groups each n=87,506; mean age 51.9 ± 19.5 years. CD n=53,077, mean age 50.4 ± 19.1; UC n=34,429, mean age 54.4 ± 20.0. No age-stratified bronchiectasis estimate reported. | Adult inpatient evidence; useful for prevalence association but not for temporal onset-age inference. |
| Liljendahl,2022 | Asthma | Danish nationwide birth cohort; children born 1991-1996 followed to age 20-25 years | Children followed from age 5. Childhood asthma defined using inhaled corticosteroid prescriptions at age 5-7 years. IBD outcome considered after age 8 years. Asthma at age 5-7 years n=18,012 (4.9%). IBD hospitalization after age 8 occurred in 2064 children; 92 with IBD before age 8 were excluded. Reverse analysis: IBD before age 10 n=118; asthma after age 10 n=15,379. | Provides clear evidence for childhood asthma exposure and subsequent IBD outcomes. |
| Peng,2015 | Asthma | Taiwan National Health Insurance Research Database; newly diagnosed adult IBD and matched non-IBD controls | Adults aged ≥20 years. IBD n=5260 and controls n=21,040. Mean age 46.59 ± 15.92 years in IBD and 46.37 ± 16.06 in controls. Age groups: 20–34, 35-49, 50-64, ≥65 years. Age was associated with asthma risk; older age categories showed higher HRs for asthma. Age group distribution was fully reported. | Adult IBD-to-asthma evidence; not childhood-onset asthma. |
| Gong,2024 | Asthma | Swedish national registers and twin cohorts; population born 1987-2014 plus twin subcohorts | National cohort included 2,907,237 individuals born 1987-2014; several twin cohorts covered birth years 1911-2012. Subanalyses by age at asthma onset were mentioned; no robust age-stratified asthma-IBD estimates used in this review. | Large life-course register evidence; age information is mainly birth-cohort based rather than onset-age strata. |
| Kisiel,2023 | Asthma | RHINE III questionnaire study in Northern Europe | IBD participants had mean age 52.2 ± 7.0 years; non-IBD 51.6 ± 7.2. UC mean age 52.4 ± 6.9; CD mean age 49.2 ± 7.4. Among 21 subjects with both age at asthma onset and IBD, 16 (76%) had asthma before IBD and 5 had IBD before asthma; mean interval between diagnoses was 17 years. | Middle-aged adult survey evidence; provides useful temporal age information for asthma onset in a subset. |
| Burisch,2019 | Asthma | Danish nationwide cohort of incident adult IBD and controls | Adults aged ≥18 years. Median age (IQR) at IBD diagnosis/index: IBD 45.8 (33.8-60.7), CD 43.9 (31.9-58.7), UC 48.8 (34.8-61.9). No age-stratified asthma estimate reported in the main extraction. | Adult IBD evidence; age profile differs from pediatric IBD/asthma studies. |
| Halling,2017 | Asthma | Danish nationwide IBD prevalence cohort with age-, sex-, and municipality-matched controls | Mean age at entry: IBD 53, UC 55, CD 49, IBD with both diagnoses 47. Controls matched. Mean age at IBD onset: IBD 42, UC 44, CD 37, both 34. No asthma-specific age-stratified estimate reported. | Adult prevalent IBD cohort; useful for age-at-IBD-onset context. |
| Haapamäki,2011 | Asthma | Finnish adult IBD survey linked to Social Insurance Institution register; matched controls | Adults ≥18 years. IBD respondents n=2831; controls n=5662. Mean age of IBD patients 44.1 ± 13.4 years. No age-stratified asthma-specific estimate reported. | Adult IBD survey; provides age and disease-duration context but limited onset-age data. |
| Sibtain,2011 | Asthma | Single-center Canadian prospective cohort of children with IBD and age-/sex-matched controls; family history study | Children ≤18 years; 108 IBD children and 108 age-/sex-matched controls. No age-stratified estimate; asthma variable was family history rather than the child’s own asthma diagnosis. | Pediatric family-history evidence; age relevance is pediatric eligibility, but not a temporal asthma-onset study. |
| Kappelman,2011 | Asthma / pediatric IBD | PharMetrics Patient-Centric Database; commercially insured US children | All individuals were <20 years old. CD cases n=737, UC cases n=488, total IBD n=1242, with matched controls n=3353. Mean age: CD cases 15.0 ± 3.2, CD controls 15.1 ± 3.4; UC cases 14.8 ± 3.7, UC controls 14.9 ± 3.7; total IBD cases 15.0 ± 3.4, controls 15.0 ± 3.5. Median age was 16 years across groups. A sensitivity analysis repeated the analysis using an age cut-off of <17 years according to the Montreal classification; no age-stratified asthma-specific estimate was reported in the main table. | Pediatric IBD evidence; age profile supports childhood/adolescent IBD context but not temporal childhood-onset asthma inference. |
| Weng,2007 | Asthma | Kaiser Permanente Northern California; IBD cases and matched controls | Mean age in 1996 was 43 years. Age distribution in IBD cases: <20 10.7%, 20-39 33.2%, 40-59 37.4%, 60–79 17.5%, ≥80 1.1%; controls matched. No age-stratified asthma estimate reported. | Mixed-age IBD cohort with age-matched controls; supports overall association but not onset-age-specific conclusions. |
| Fenta,2010 | Asthma | Rochester Epidemiology Project / Olmsted County case-control study | IBD cases identified from 1964-1983; controls age-matched. Mean age at IBD diagnosis/index reported as 33.8 years; median IBD index age 28.5 years. No formal age-stratified estimate for asthma-IBD in the extracted analysis. | Historic population-based case-control evidence; younger adult IBD onset on average. |
| Yun,2012 | Asthma | Rochester asthma cohort and matched controls | Asthma onset/index mean age 15.1 years; median 5 years (IQR 1-22), indicating many childhood-onset asthma cases. Age-stratified incidence rates were presented for children and adults, although CIs were wide. | Important asthma-to-IBD evidence with predominantly childhood-onset asthma profile. |
| Krishna,2019 | Asthma | UK THIN general practice database | Asthma cohort n=1,049,868; mean baseline age 35.61 ± 21.26 years. Mean age at allergy/asthma diagnosis 25.09 ± 21.95. Age distribution ranged from 0-9 through ≥70 years. Age subgroup analyses were performed in supplementary analyses, but detailed numeric estimates were not used for pooling here. | Large all-age primary-care cohort; captures both childhood and adult asthma but pooled estimate is not onset-age-specific. |
| Kuenzig,2017 | Asthma / CD | Alberta IBD database; pediatric, young-adult, and older-adult IBD diagnosis groups; population controls | Age at IBD diagnosis/index: CD median 36 (IQR 22-51), UC 39 (25-54), controls 44 (28-59). Age groups: A1 3-16, A2 17–40, A3 >40 years. Age effect modification: no modification for CD; UC showed age effect modification (P=0.0103). UC ORs by age: ≤16 1.49 (1.08-2.07), 17–40 1.05 (0.86-1.26), >40 1.57 (1.31-1.89). | Key study for onset-age/age-at-IBD diagnosis review. It supports age-related heterogeneity particularly for UC. |
| Virta,2013 | Asthma / CD | Finnish national reimbursement registers; pediatric IBD cases and matched controls | Pediatric IBD: 233 CD and 362 UC cases; matched controls n=2380. Median age at IBD diagnosis: CD 10.9 (IQR 7.7-13.0), UC 9.5 (5.4-12.2), controls 10.2 (6.1-12.6). Asthma diagnosed beyond age 3 showed stronger association with CD than asthma diagnosed younger. | Important pediatric evidence; directly addresses childhood asthma timing before pediatric IBD. |
| Gearry,2012 | Asthma / CD | Canterbury, New Zealand case-control study; CD, UC, and controls | Cases and controls were aged 20–89 years. Age groups reported. CD cases: 20–29 n=127, 30–39 n=166, 40–49 n=121, 50–59 n=83, >59 n=141. UC cases: 57, 111, 147, 136, 202. Controls: 72, 157, 127, 96, 147. Age at recruitment was included in multivariable models; age categories 20–34, 35-49, 50-64, ≥65 were used. | Adult/mixed-age case-control evidence; useful for age composition but not onset-age-specific asthma conclusions. |
| Lo,2021 | Asthma / CD | Nurses’ Health Study II; female nurses | Women aged 25-42 years at enrollment in 1989; 1995 baseline. Age at baseline by number of immune-mediated diseases: 40.1 ± 4.7, 40.6 ± 4.6, 40.9 ± 4.5 years. No asthma-specific age-stratified estimate used in this review. | Adult female cohort; asthma/immune-mediated disease history precedes adult IBD in many participants. |
| Soh,2021 | Asthma / CD | Korean National Health Insurance Service health-checkup cohort; adults ≥20 years | Adults aged ≥20. Baseline atopic disease cohort n=1,557,435 and non-atopic n=8,366,086. Age groups in atopic group: 20-39 23.7%, 40–64 56.7%, ≥65 19.6%; non-atopic: 32.6%, 55.5%, 11.9%. Subgroup analyses by age <65 vs ≥65 reported. For asthma and incident CD: <65 HR 1.53 (1.075-2.176); ≥65 HR 1.57 (0.929-2.643). | Adult atopic-disease cohort with age subgrouping; not childhood asthma. |
| Ghersin,2020 | Asthma / UC | Israeli Defense Forces pre-enlistment adolescent cohort | Adolescents at mandatory medical evaluation; median age at evaluation 17.1 years (IQR 16.7-17.3). Median age at IBD diagnosis 15 years (IQR 12.5-17). No age-stratified asthma estimate; multivariable models included age at diagnosis. | Adolescent IBD evidence; important for non-adult age profile but not for temporal childhood-onset asthma inference. |
| Boneberger,2012 | Asthma / UC | Chilean hospital-based case-control study of UC and controls | UC cases age range 6-45 years. Mean age UC 27.4 ± 8.3; controls 26.2 ± 8.2. Age adjusted as <26 versus ≥26 years. No age-stratified asthma estimate; asthma OR adjusted for age and sex. | Small mixed pediatric/adult UC sample; age range should be reported because it differs from large adult registry studies. |
| Wasielewska,2019 | Asthma / UC | Polish pediatric IBD case-control questionnaire study | Children with IBD n=60 and controls n=60. Current age: CD 15.0 ± 3.08, UC 14.3 ± 3.89, IBD overall 14.72 ± 3.41, controls 14.85 ± 2.92 years. Range IBD 4.75-19.25 years. No age-stratified asthma estimate; Paris classification age groups used. | Pediatric/adolescent IBD evidence; useful for onset-age narrative but estimates are imprecise because of small sample size. |
| Myrelid,2004 | Asthma / CD | Swedish CD questionnaire study with population controls | CD patients aged 18-50 years; controls in same age range and selected from same region. No age-stratified asthma estimate. | Young-to-middle adult CD sample; not pediatric and not older-adult. |
| OLD: obstructive lung diseases; IBD: inflammatory bowel disease; COPD: chronic obstructive pulmonary disease; CD: Crohn’s disease; UC: ulcerative colitis; HR: hazard ratio; OR: odds ratio; IQR: interquartile range; PY: person-years; NHIS: National Health Insurance Service; THIN: The Health Improvement Network; RHINE: Respiratory Health in Northern Europe; UK: United Kingdom; US: United States. | | | | |

# Supplementary Figures


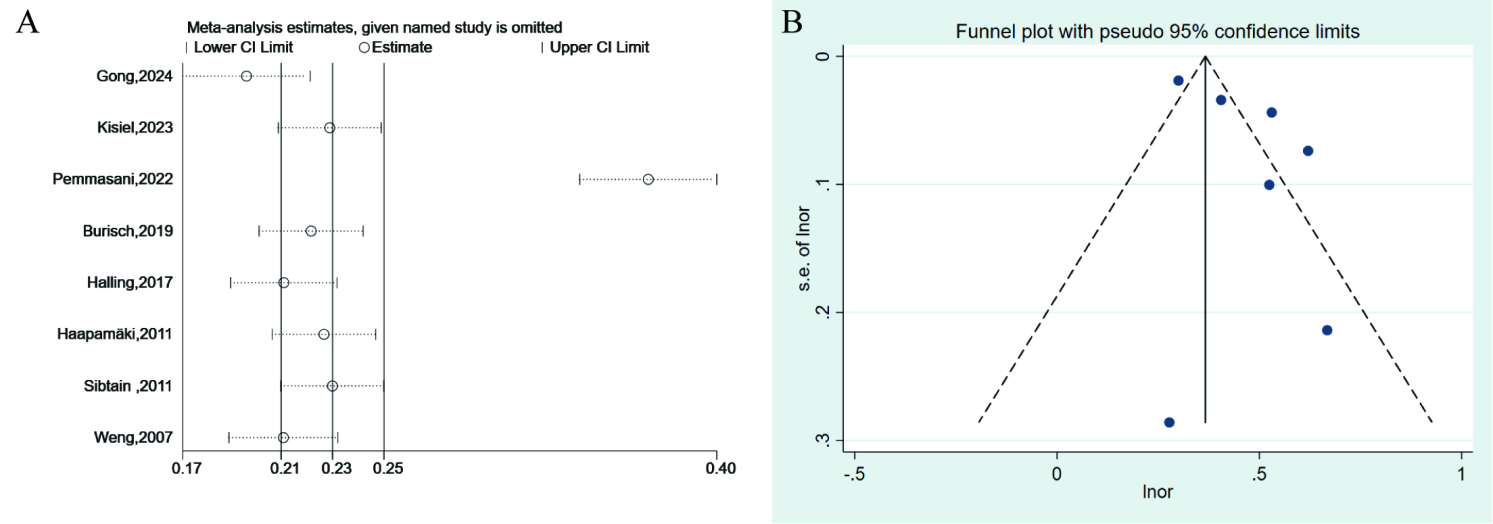


Supplementary Figure 1. Sensitivity analysis and funnel plot for the association between IBD and subsequent asthma based on OR estimates.

(A) Sensitivity analysis of the association between IBD and subsequent asthma.

(B) Funnel plot assessing publication bias for the association between IBD and subsequent asthma.


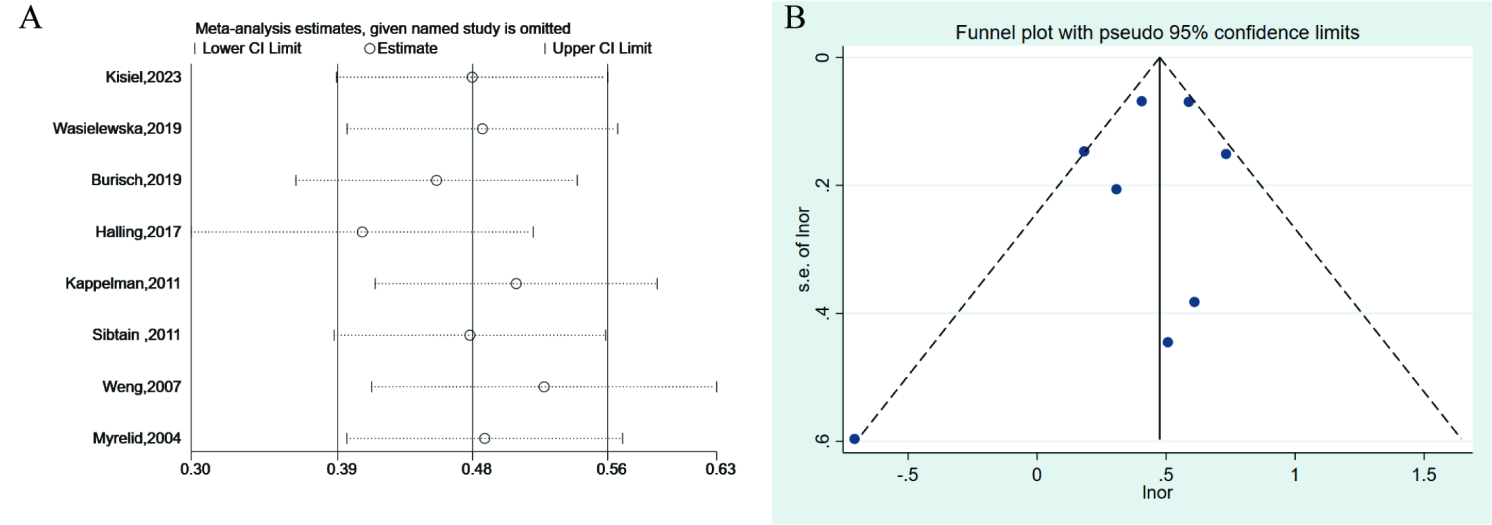


Supplementary Figure 2. Sensitivity analysis and funnel plot for the association between Crohn’s disease and subsequent asthma based on OR estimates.

(A) Sensitivity analysis of the association between Crohn’s disease and subsequent asthma.

(B) Funnel plot assessing publication bias for the association between Crohn’s disease and subsequent asthma.


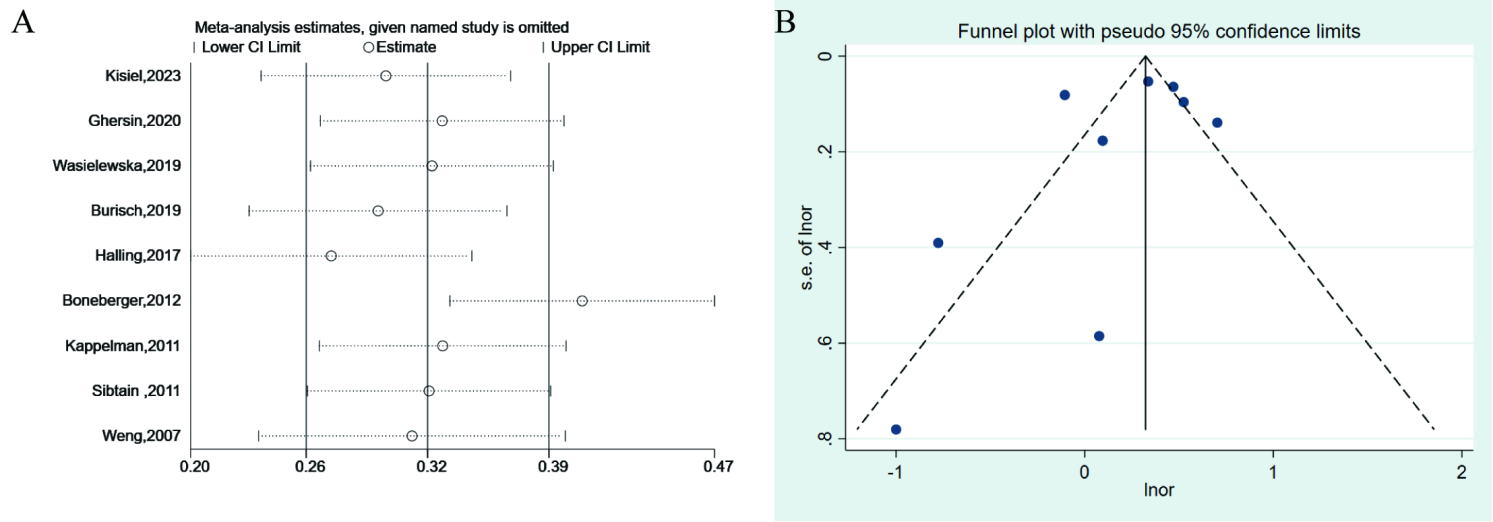


Supplementary Figure 3. Sensitivity analysis and funnel plot for the association between ulcerative colitis and subsequent asthma based on OR estimates.

(A) Sensitivity analysis of the association between ulcerative colitis and subsequent asthma.

(B) Funnel plot assessing publication bias for the association between ulcerative colitis and subsequent asthma.
